# Supplementary material for: Unravelling and reconstructing the biosynthetic pathway of bergenin
Source: Nat Commun. 2024 Apr 26;15:3539. doi: 10.1038/s41467-024-47502-2 (PMC11053098; doi:10.1038/s41467-024-47502-2)
Supplement: Supplementary file 1 — Supplementary Information [file 41467_2024_47502_MOESM1_ESM.pdf]

Unravelling and reconstructing the biosynthetic pathway of bergenin

Yan *et al.*

Supplementary Method 1. Isolation and purification of norbergenin (6)

The dried aerial of *Ardisia japonica* (2.5 Kg) were extracted with 75% EtOH under reflux to give the extract after solvent removal at reduced pressure. The extract was partitioned with H_2O, extracted successively with CH_2Cl_2 and *n*-BuOH. A portion (100 g) of the *n*-BuOH-soluble fraction (110 g) was subjected to silica gel column chromatography with CH_2Cl_2-MeOH of increasing polarity to give 12 fractions. Fr.8 (13.4 g) was separated by reverse-phase C_{18} column chromatography with MeOH-H_2O (1: 19) to give norbergenin (880 mg). ^{1}H NMR (400 MHz, DMSO-d_6) δ: 9.50 (1H, s, HO-5), 9.33 (1H, s, HO-4), 8.26 (1H, s, HO-3), 6.96 (1H, s, H-6), 5.60 (1H, d, J = 5.6 Hz, HO-3′), 5.40 (1H, d, J = 5.6 Hz, HO-4′), 4.94 (1H, d, J = 9.6 Hz, H-1′), 4.89 (1H, t, J = 4.8 Hz, HO-6′), 3.92 (1H, dd, J = 10.4, 10.0 Hz, H-2′), 3.84 (1H, brd, J = 11.2 Hz, H-6′a), 3.64 (1H, ddd, J = 9.2, 8.8, 4.4 Hz, H-3′), 3.57 (1H, ddd, J = 7.6, 7.6, 2.0 Hz, H-5′), 3.42 (1H, dd, J = 11.2, 8.0 Hz, H-6′b), 3.20 (1H, ddd, J = 9.2, 8.4, 4.4 Hz, H-4′); ^{13}C NMR (100 MHz, DMSO-d_6) δ: 163.8 (C-7), 145.9 (C-5), 142.4 (C-3), 139.6 (C-4), 116.0 (C-1), 112.7 (C-2), 109.3 (C-6), 81.6 (C-5′), 79.8 (C-3′), 72.0 (C-1′), 73.7 (C-2′), 70.8 (C-4′), 61.2 (C-6′) [1].

Supplementary Method 2. Preparing 4-*O*-methyl gallic acid 2-*C*-*β*-D-glycoside (4) through bergenin (1) hydrolysis

A total of 1 M aqueous solution of sodium hydroxide (2 mL) was added to bergenin (99 mg), and the mixture was stirred for three hours at room temperature. Then, the reaction mixture was neutralized by adding 1 M aqueous solution of hydrochloric acid (2 mL). The reaction mixture was subjected to semipreparative reversed-phase HPLC with MeOH-H_2O (1: 9) to afford 4-*O*-methyl gallic acid 2-*C*-*β*-D-glycoside (82 mg, this compound is very easily esterified to form bergenin again, try to keep low temperature during preparation, and fraction from HPLC was frozen drying). ^{1}H NMR (400 MHz, DMSO-d_6) δ: 9.40 (1H, s, HO-5), 8.67 (1H, s, HO-3), 6.68 (1H, s, H-6), 5.09 (1H, d, J = 9.6 Hz, H-1′), 4.95 (1H, d, J = 6.0 Hz, HO-4′), 4.94 (1H, d, J = 5.6 Hz, HO-3′), 4.56 (1H, t, J = 5.2 Hz, HO-6′), 3.71 (3H, s, H-8), 3.65 (1H, dd, J = 11.2, 5.2 Hz, H-6′a), 3.56 (1H, dd, J = 11.2, 4.8 Hz, H-6′b), 3.51 (1H, dd, J = 9.6, 8.6 Hz, H-2′), 3.20–3.25 (3H, m, H-3′–5′); ^{13}C NMR (100 MHz, DMSO-d_6) δ: 169.4 (C-7), 150.1 (C-3), 149.3 (C-5), 137.9 (C-4), 128.1 (C-1), 116.3 (C-2), 108.8 (C-6), 80.9 (C-5′), 78.3 (C-3′), 76.7 (C-1′), 72.1 (C-2′), 69.3 (C-4′), 60.4 (C-6′), 59.7 (C-8).

Supplementary Method 3. Determination of bergenin contents in different organs of *Ardisia japonica*

Fresh plants were washed by water, then were dried in an oven at 42 °C. Dry leaves, stems, and rhizomes after weighing were grounded in a mortar respectively, the dried powder of different organs was extracted (30 min × 3) in an ultrasonic bath with 100 mL MeOH, and the MeOH extract was concentrated under vacuum at 42 °C. The extract of different organs was dissolved with methanol and diluted to 5 mL with a volumetric flask, the solution was diluted 100 times again, then was filtered and used for HPLC analysis. The above steps were repeated twice again. The quantification of bergenin was carried out by interpolation of the peak areas obtained by HPLC with the standard curve. The standard curve was prepared by HPLC analysis of bergenin. The HPLC condition was as follow: 5–25% methanol linear gradient (30 min) in 0.1% formic acid aqueous solution.

Supplementary Method 4. Quantitative analysis of bergenin, GA, 3,4-DHBA, 4-OMGA, 4-OMGA-Glc

A total of 12.8 mg (6.4 mg for 4-OMGA-Glc) of sample was dissolved in 10 mL methanol. 1 mL of the above solution was diluted to 2 mL to obtain a solution with a concentration of 640 μg L^{-1}. Likely, 320 μg L^{-1}, 160 μg L^{-1}, 80 μg L^{-1}, 40 μg L^{-1}, 20 μg L^{-1}, 10 μg L^{-1}, 5 μg L^{-1} was obtained by repeating the above operation successively. Samples of different concentrations were analyzed by HPLC-MS, the injection volume was 10 μL. Three parallel assays were routinely conducted. Standard curve was created basing on the relationship between peak area and injection quality.

Supplementary Method 5. Effects of pH, temperature and divalent metal ions on enzymatic activity of AjCGT1

To investigate the optimal pH, the enzymatic reaction was performed in various reaction buffers with pH values in the range of 5.0–6.0 (citric acid-sodium citrate buffer), 6.0–7.0 (Na_2HPO_4-NaH_2PO_4 buffer), and 7.0–10.0 (Tris-HCl buffer), 10.0–11.0 (Na_2CO_3-$NaHCO_3$ buffer) at 40 °C for

2 h using gallic acid (**2**) as substrate. The enzymatic reaction was performed in various reaction buffers with pH values in the range of 5.0–6.0 (citric acid-sodium citrate buffer), 6.0–8.0 (Na_2HPO_4-NaH_2PO_4 buffer), and 8.0–9.0 (Tris-HCl buffer), 9.0–11.0 (Na_2CO_3-$NaHCO_3$ buffer) at 30 °C for 10 min using 4-*O*-methyl gallic acid (**3**) as substrate. To assay the optimal reaction temperature, the reactions were incubated at different temperatures (20–60 °C) for 2 h (**2**) or 10 min (**3**). To investigate the effects of divalent metal ions on AjCGT1 activity, $BaCl_2$, $CaCl_2$, $CuCl_2$, $FeCl_2$, $SnCl_2$, $SrCl_2$, $ZnCl_2$, $MgCl_2$, $MnCl_2$, $NiSO_4$, and EDTA were used individually in the final concentration of 5 mM, The reactions were performed at 35 °C for 2 h (**2**) or 40 °C for 10 min (**3**). Tris-HCl buffer (pH 8.0) was used for **2**, Na_2HPO_4-NaH_2PO_4 buffer (pH 8.0) were used for **3**. All incubations were performed with UDP-Glc (0.8 mM) as a donor and **2** or **3** as an acceptor (0.4 mM), and 20 µg purified AjCGT1. All reaction buffers were 50 mM. Aliquots were quenched by adding ice cold methanol and centrifuged at 15,000 g for 30 min, then supernatants were analyzed by HPLC-MS. Three parallel assays were routinely conducted.

Supplementary Method 6. Effects of pH, temperature and metal ions on enzymatic activity of AjOMT2

To investigate the optimal pH, the enzymatic reaction was performed in various reaction buffers with pH values in the range of 5.0–6.0 (citric acid-sodium citrate buffer), 6.0–7.0 (Na_2HPO_4-NaH_2PO_4 buffer), and 7.0–9.0 (Tris-HCl buffer), 9.0–11.0 (Na_2CO_3-$NaHCO_3$ buffer) at 37 °C for 6h using GA (**2**) or norbergenin (**6**) as substrate. To assay the optimal reaction temperature, the reactions were incubated at different temperatures (20–60 °C) for 30 min (**2** or **6**). To test the effects of metal ions on AjCGT1 activity, KCl, $CaCl_2$, $FeCl_2$, $ZnCl_2$, $MgCl_2$, $CuCl_2$, $MnCl_2$, $BaCl_2$, $CoCl_2$, $NiSO_4$, and EDTA were used individually in the final concentration of 5 mM, The reactions were performed at 40 °C for 30 min (**2** or **6**). Na_2HPO_4-NaH_2PO_4 buffer (pH 7.0) was used for **2**, Tris-HCl buffer (pH 7.0) were used for **6**. All determinations were performed with SAM (0.8 mM) as a donor, **2** or **6** as an acceptor (0.4 mM), $MgCl_2$ (1 mM), 50 µg purified AjOMT2. All reaction buffers were 50 mM. Aliquots were quenched by adding ice cold methanol and centrifuged at 15,000 g for 30 min, and the supernatants were analyzed by HPLC-MS. Three parallel assays were routinely conducted.

Supplementary Method 7. Determination of kinetic parameters of AjCGT1 and AjOMT2

For kinetic studies of AjCGT1, reaction mixture contained 50 mM Na_2HPO_4-NaH_2PO_4 buffer (pH 8.0), UDP-glc (0.8 mM), 10 μg purified AjGT1 and varying concentration (20 μM, 40 μM, 100 μM, 200 μM, 400 μM, 800 μM, 1000 μM, 2500 μM) of 4-OMGA (**3**) at 40 °C in a total volume of 100 μL for reaction in 10 min. For kinetic studies of AjOMT2, assays were performed in a final volume of 100 μL 50 mM Na_2HPO_4-NaH_2PO_4 buffer (pH 7.0) for GA (**2**) or Tris-HCl buffer (pH 7.0) for norbergenin (**6**), and contained 10 μg purified AjOMT3, 2 μL SAM (40 mM), 2.5 μL $MgCl_2$ (40 mM) and varying **2** or **6** concentrations (0.04–4 mM), respectively. The mixtures were incubated at 40 °C for 10 min. Aliquots were quenched by adding ice cold methanol and centrifuged at 15,000 g for 30 min, and the supernatants were analyzed by HPLC-MS. All experiments were performed in triplicate.

Supplementary Method 8. Extraction, isolation and purification of bergenin (1) from cultures of bioreactor

The pH value of the cultures from the 3 L bioreactor was adjusted to pH 0.1 and extracted with equivalent volume of *n*-BuOH for three times. The organic layer was evaporated under reduced pressure to give a gummy residue (39 g). A portion of the gummy residue (5.97 g) was separated by reverse-phase C_{18} column chromatography eluted successively with MeOH-H_2O (1: 19, 1: 9, 2: 8, 3: 7, *v*/*v*) to give four fractions. Bergenin crystallized in the 3rd fraction (MeOH-H_2O, 2: 8, *v*/*v*), and recrystallization of the 3rd fraction in MeOH yielded pure bergenin (376 mg).

Supplementary Table 1. Contents of bergenin in different organs of *A. japonica.*

Organ	Bergenin content (% of dry weight, *n*=3)
Rhizome	0.080±0.005
Stem	0.088±0.009
Leaf	0.280±0.015

Supplementary Table 2. The differential expression of two CGT candidate genes from *A. japonica.*

Gene name	Gene ID	Length (bp)	FPKM			Annotation
			Rhizome	Stem	Leaf	
AjCGT1	*CL7566.contig1*	1353	64.08	65.54	196.93	2-hydroxyflavanone CGT
AjCGT2	*Unigene14257*	822	17.41	13.14	70.02	2-hydroxyflavanone CGT

Supplementary Table 3. The specific activity of AjCGT1, AjCGT2 and other reported CGTs.

Enzymes	Substrates	specific activity (pkat/mg)[a]
AjCGT1	4-OMGA (**3**)	3667
AjCGT2	4-OMGA (**3**)	1871
AjCGT1	GA (**2**)	868
AjCGT2	GA (**2**)	370
AjCGT1	phloretin	13827
OsCGT[2]	phloretin	587
FcCGT[3]	phloretin	34200
CuCGT[3]	phloretin	6600

[a] The reaction was performed with 0.4 mM acceptors, 0.8 mM UDP-Glc and 10 µg purified AjCGTs in a total volume of 100 µL and the mixture was incubated at 40 °C for 10 min. For the acceptor of 4-OMGA, the reaction was performed in 50 mM Na_2HPO_4-NaH_2PO_4 buffer (pH 8.0); while for GA, the reaction was performed in 50 mM Tris-HCl buffer (pH 7.0).

Supplementary Table 4. The differential expression of ten OMT candidate genes from *A. japonica*.

Gene	Length (bp)	PFKM			Annotation
		Rhizome	Stem	Leaf	
AjOMT1	738	68.83	206.07	75.6	caffeoyl-CoA OMT
AjOMT2	717	3.42	4.12	14.68	caffeoyl-CoA OMT
AjOMT3	717	7.20	7.79	19.96	caffeoyl-CoA OMT
AjOMT4	1095	3.49	4.21	21.58	caffeic acid 3-OMT
AjOMT5	1056	14.19	13.68	77.37	trans-resveratrol di-OMT
AjOMT6	1095	60.33	57.41	200.39	caffeic acid 3-OMT
AjOMT7	1074	17.14	6.99	93.84	trans-resveratrol di-OMT
AjOMT8	1068	14.85	5.33	4.87	trans-resveratrol di-OMT
AjOMT9	1074	217.82	56.06	1.15	scoulerine 9-OMT
AjOMT10	741	24.76	55.51	1.31	caffeoyl-CoA OMT

Supplementary Table 5. The kinetic parameters of OMTs with different substrates.

Enzymes	Substrates	K_m (μM)[a]	K_{cat} (s^{-1})[a]	K_{cat}/K_m ($M^{-1}\cdot s^{-1}$)[a]
AjOMT2	GA (**2**)	72.0	1.8×10^{-2}	250
AjOMT2	norbergenin (**6**)	493.6	1.9×10^{-2}	38.5
CrOMT1[4]	myricetin	23.8	6.7×10^{-4}	28.0
CrOMT1[4]	tricetin	0.6	7.6×10^{-4}	1387
VpOMT4[5]	tricetin	158	1.11×10^{-2}	70
VpOMT5[5]	tricetin	109	9.13×10^{-2}	838

[a] The kinetic parameters of AjOMT2 were measured at 40 °C and pH 7.0 (50 mM Tris-HCl buffer).

Supplementary Table 6. Strains used in this study.

Strains	Description	Source
S1	BL21 (DE3) with pET-*pobA**-UbiC*	This study
S2	BL21 (DE3) with pCDF-*pobA**-UbiC*	This study
S3	BL21 (DE3) with pACYC-*pobA**-UbiC*	This study
E1	BL21 (DE3) with pET-*AjOMT2-AjCGT1*	This study
E2	BL21 (DE3) with pCDF-*AjOMT2-AjCGT1*	This study
E3	BL21 (DE3) with pACYC-*AjOMT2-AjCGT1*	This study
A1	BL21 (DE3) with pET-*pobA**-UbiC* and pACYC-*AjOMT2-AjCGT1*	This study
A2	BL21 (DE3) with pET-*pobA**-UbiC* and pCDF-*AjOMT2-AjCGT1*	This study
A3	BL21 (DE3) with pCDF-*pobA**-UbiC* and pET-*AjOMT2-AjCGT1*	This study
A4	BL21 (DE3) with pCDF*pobA**-UbiC* and pACYC-*AjOMT2-AjCGT1*	This study
A5	BL21 (DE3) with pACYC-*pobA**-UbiC* and pET-*AjOMT2-AjCGT1*	This study
A6	BL21 (DE3) with pACYC-*pobA**-UbiC* and pCDF-*AjOMT2-AjCGT1*	This study
M1	BL21 (DE3) with pET-*AjOMT2*-AjCGT1*	This study
M2	BL21 (DE3) with pET-*AjOMT2-AjCGT1**	This study
M3	BL21 (DE3) with pET-*AjOMT2*-AjCGT1**	This study
Y1	BL21 (DE3) with pET-$AjOMT2^{opt}$-*AjCGT1*	This study
Y2	BL21 (DE3) with pET-*AjOMT2*-$AjCGT1^{opt}$	This study
Y3	BL21 (DE3) with pET-$AjOMT2^{opt}$-$AjCGT1^{opt}$	This study
Y4	BL21 (DE3) with pET-$AjOMT2^{*opt}$-$AjCGT1^{opt}$	This study
Y5	BL21 (DE3) with pET-$AjOMT2^{opt}$-$AjCGT1^{*opt}$	This study
D1	BL21 (DE3) with pET-$AjOMT2^{*opt}$-$AjCGT1^{opt}$ and pCDF-*pobA**-UbiC*	This study
D2	BL21 (DE3) with pET-$AjOMT2^{*opt}$-$AjCGT1^{opt}$ and pCDF-*pobA**-UbiC* and pACYCDuet-1	This study
D3	BL21 (DE3) with pET-$AjOMT2^{*opt}$-$AjCGT1^{opt}$ and pCDF-*pobA**-UbiC* and pACYC-*metK*	This study
D4	BL21 (DE3) with pET-$AjOMT2^{*opt}$-$AjCGT1^{opt}$ and pCDF-*pobA**-UbiC* and pACYC-*mtn-luxS*	This study
D5	BL21 (DE3) with pET-$AjOMT2^{*opt}$-$AjCGT1^{opt}$ and pCDF-*pobA**-UbiC* and pACYC-*galU-pgm*	This study

Supplementary Table 7. HPLC methods in this study.

Method	Solvent A	Solvent B	Gradient	Analysis purpose
A	Water containing 0.1% formic acid	MeOH	5–15% B, 20 min; 15–100% B, 5 min 100% B, 5 min	Functional Characterization of the AjCGTs (**2** was the substrate)
B	Water containing 0.1% formic acid	MeOH	5–45% B, 20min; 45–100% B, 5 min 100% B, 5 min	Functional Characterization of the AjCGTs (**3** was the substrate)
C	Water containing 0.1% formic acid	ACN	5–15% B, 15 min; 15–45% B, 15 min; 45–100% B, 5 min; 100% B, 5 min	Functional Characterization of the AjOMTs (**2** was the substrate)
D	Water containing 0.1% formic acid	ACN	5–45% B, 30 min; 45–100% B, 5 min; 100% B, 5 min	Functional Characterization of the AjOMTs (**6** was the substrate)
E	Water containing 0.1% formic acid	ACN	1–10% B, 19 min; 10–15% B, 1 min; 15–45% B, 15 min; 45–100% B, 1 min; 100% B, 5 min	*De novo* biosynthesis of bergenin precursor 4-OMGA-Glc
F	Water containing 0.1% formic acid	MeOH	5–65% B, 30 min; 65–100% B, 5 min 100% B, 5 min	maclurin as the substrate
G	Water containing 0.1% formic acid	MeOH	15–100% B, 30 min; 100% B, 5 min	phloretin, apigenin, 2-hydoroxynaringenin, 5, 7-dihydroxycoumarin as the substrate

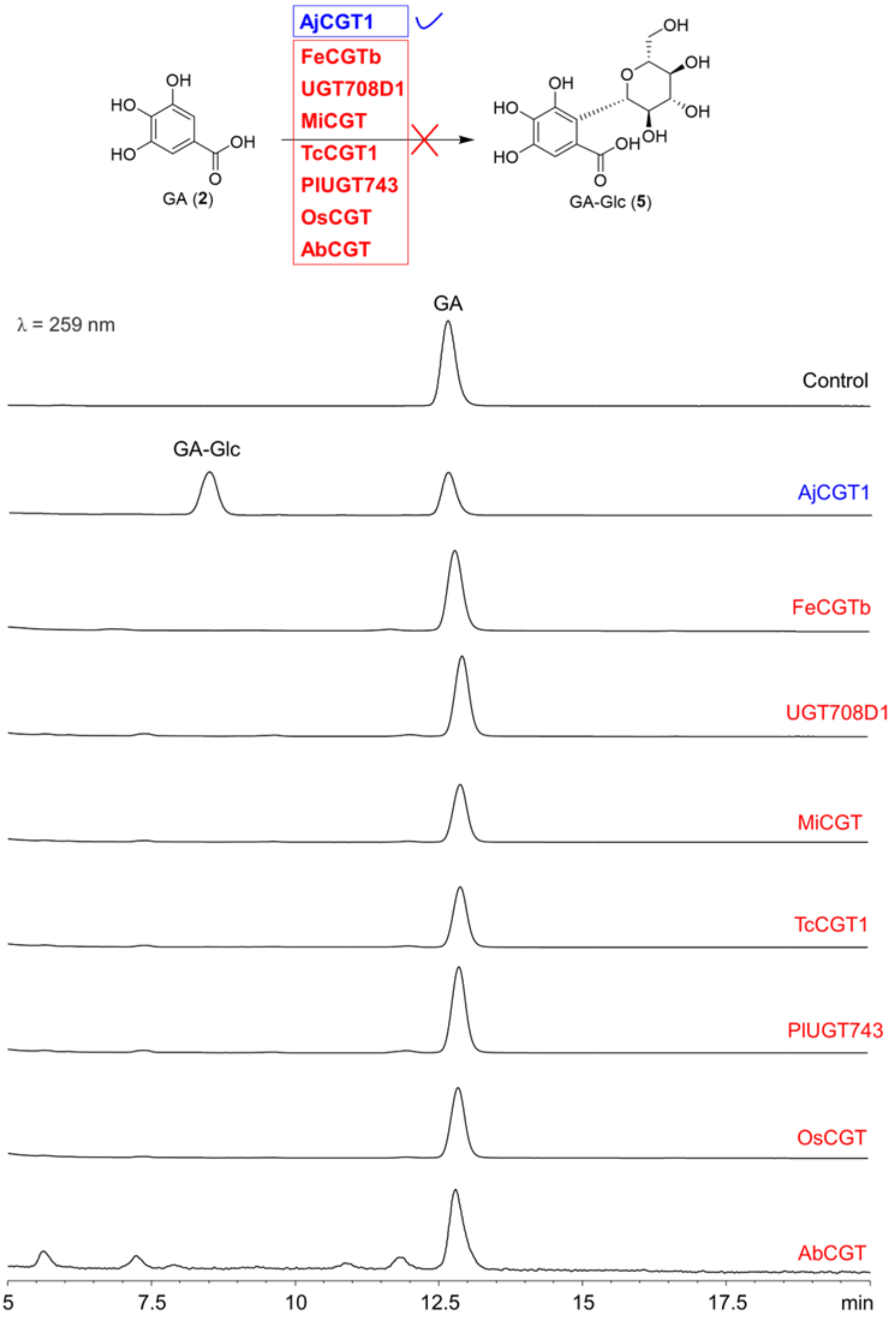

Supplementary Fig. 1. Probing the *C*-glycosylation activities of the representative reported CGTs to GA (2). All these seven CGTs showed no *C*-glycosylation activity to GA (**2**). The Genebank accession numbers of the seven CGTs used for analysis are as follows: FeCGTb (AB909376); UGT708D1 (LC003312); MiCGT (KT200208); TcCGT1 (MK644229); PlUGT43 (A0A172J2G3.2); OsCGT (FM179712); AbCGT (MN747405). The *in vitro* assays of CGTs (in a total volume of 100 μL) consisted of 50 mM Tris-HCl buffer (pH 7.0), 0.8 mM UDP-Glc, 0.4 mM GA and 50 μg purified recombinant enzyme, and the mixture was incubated at 40 °C for 1 h.

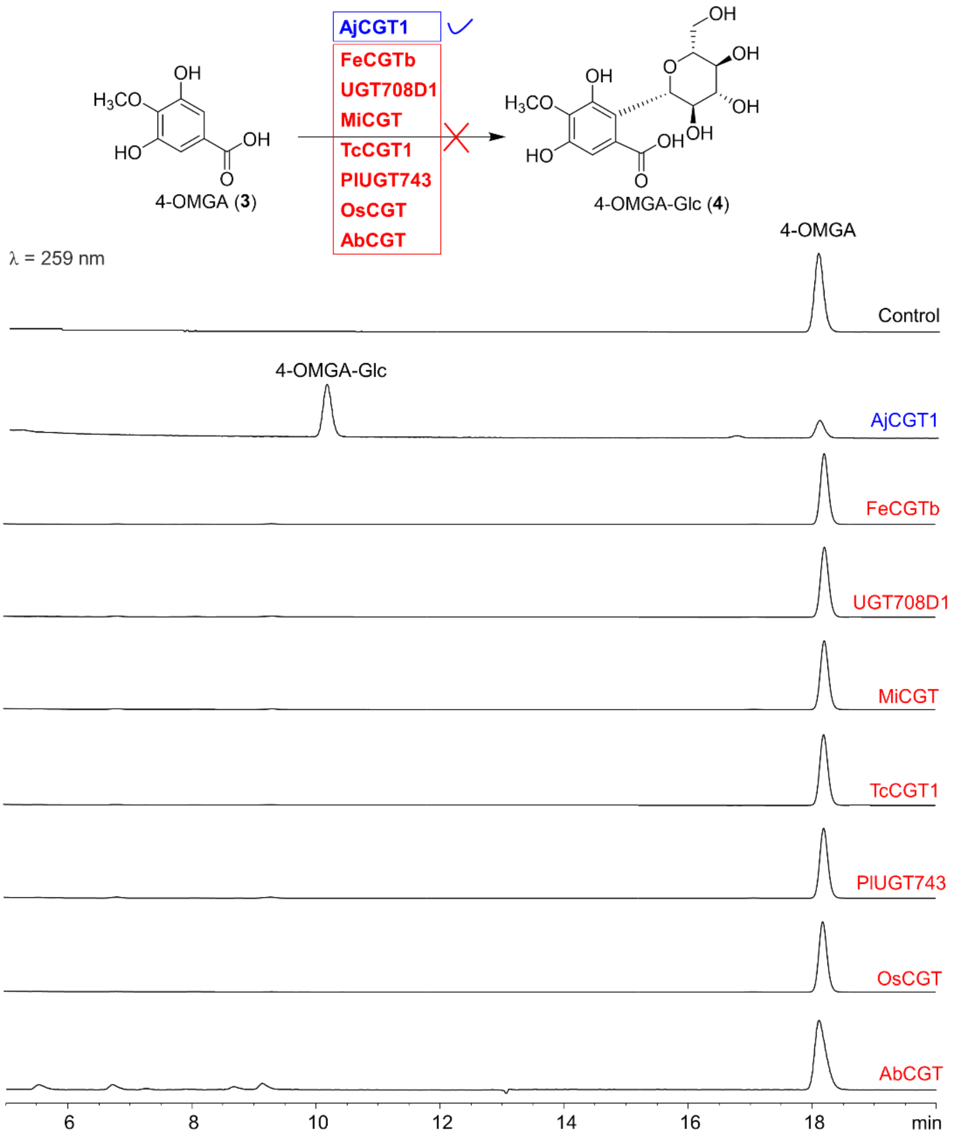

Supplementary Fig. 2. Probing the *C*-glycosylation activities of the representative reported CGTs to 4-OMGA (3). All these seven CGTs showed no *C*-glycosylation activity to 4-OMGA (**3**). The Genebank accession numbers of the seven CGTs used for analysis are as follows: FeCGTb (AB909376); UGT708D1 (LC003312); MiCGT (KT200208); TcCGT1 (MK644229); PlUGT43 (A0A172J2G3.2); OsCGT (FM179712); AbCGT (MN747405). The *in vitro* assays of CGTs (in a total volume of 100 μL) consisted of 50 mM Tris-HCl buffer (pH 7.0), 0.8 mM UDP-Glc, 0.4 mM 4-OMGA and 50 μg purified recombinant enzyme, and the mixture was incubated at 40 °C for 1 h.

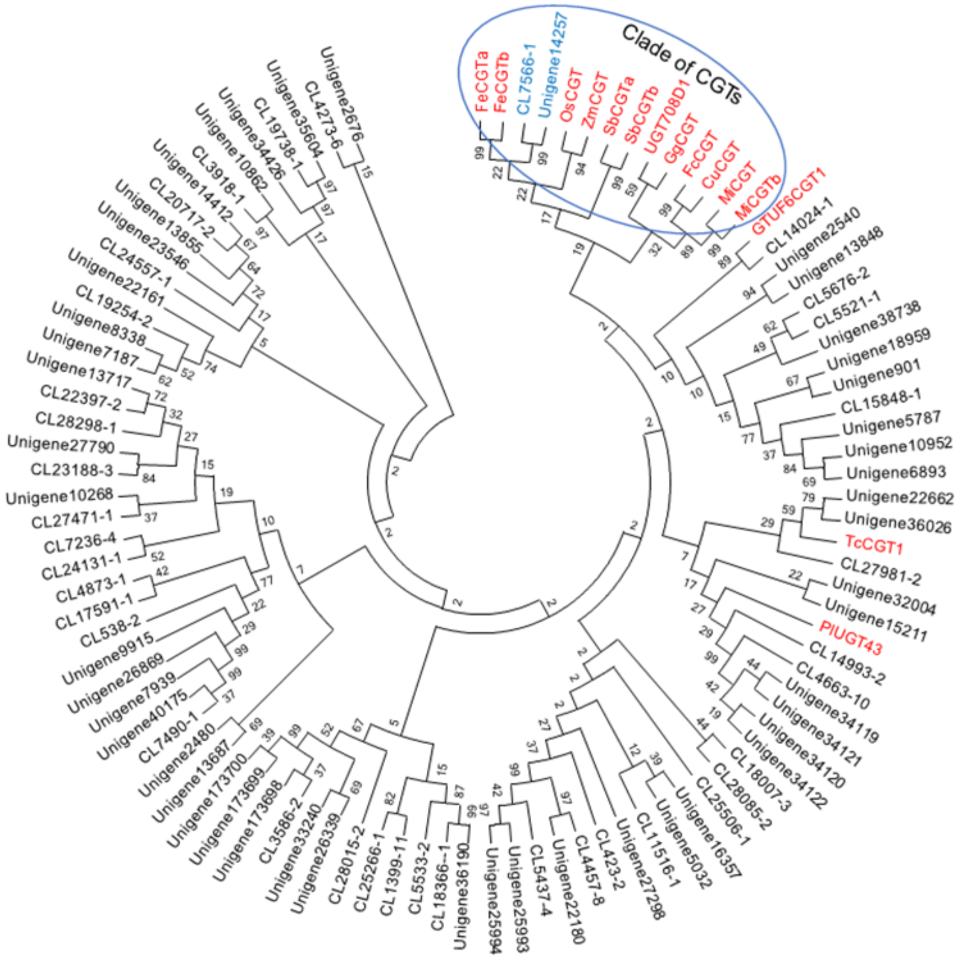

Supplementary Fig. 3. Phylogenetic analysis of AjGTs from *A. japonica* with higher plant CGTs. The phylogenetic tree was constructed using the neighbor-joining method in MEGA 7.0. The clade of CGTs including CL7566-1 (AjCGT1), Unigene14257 (AjCGT2) is circled in blue, previously characterized CGTs are indicated in red. CLxxxx-x represent CLxxxx.contigx. the Genebank accession numbers of CGTs used for analysis are as follows: FeCGTa (AB909375); FeCGTb (AB909376); OsCGT (FM179712); ZmCGT (NP_001132650); SbCGTa (MK894443); SbCGTb (MK894444); UGT708D1 (LC003312); GgCGT (MH998596); FcCGT (LC131333); CuCGT (LC131334); MiCGT (KT200208); MiCGTb (KT989668); GTUF6CGT1 (AB985754); TcCGT1 (MK644229); PlUGT43 (A0A172J2G3.2).

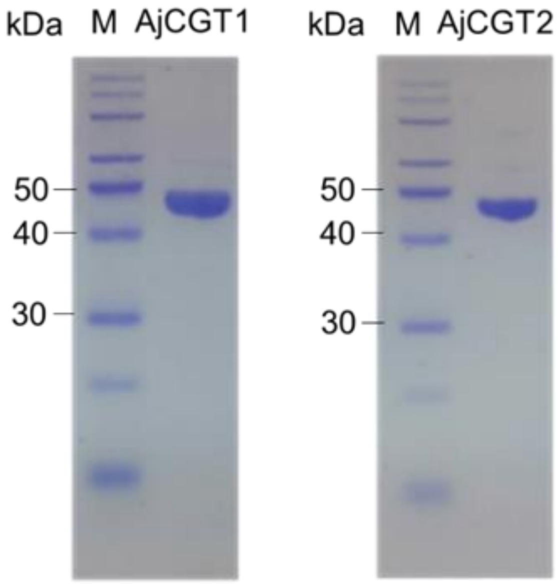

Supplementary Fig. 4. SDS-PAGE of recombinant His$_6$-AjCGT1 and His$_6$-AjCGT2 purified by affinity chromatography. Lane M: Protein Marker; Lane AjCGT1: His-tagged AjCGT1 (predicted M.W., 49.8 kDa) purified on Ni Sepharose; Lane AjCGT2: His-tagged AjCGT2 (predicted M.W., 49.7 kDa) purified on Ni Sepharose column chromatography. Source data are provided as a Source Data file.

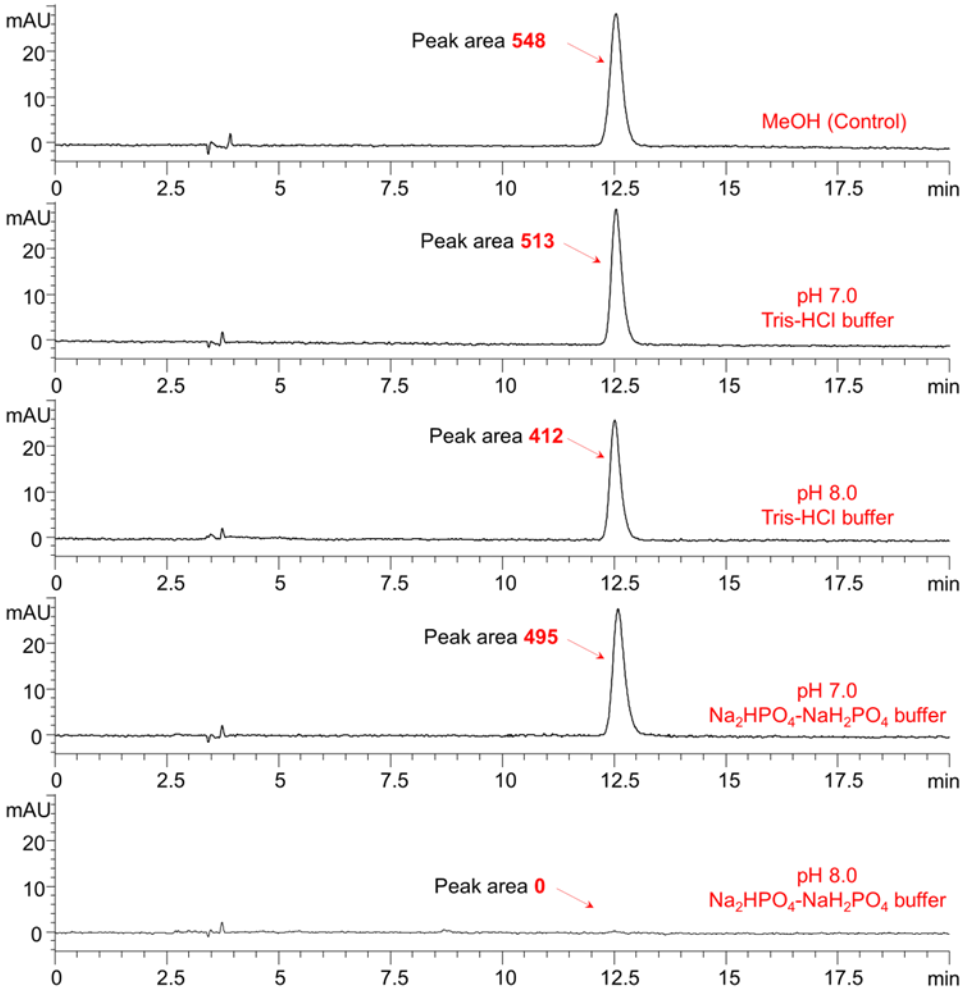

Supplementary Fig. 5. Investigation of the stability of GA (2) in different buffers. GA (**2**) was added into MeOH (control) and the corresponding buffers for 10 minutes to evaluate its stability. HPLC analysis results showed that the peak areas of GA (**2**) in MeOH, pH7.0 Tris-HCl, pH8.0 Tris-HCl, pH7.0 Na_2HPO_4-NaH_2PO_4 and pH8.0 Na_2HPO_4-NaH_2PO_4 buffer were 548 (control), 513, 412, 495 and 0, respectively. These results indicated GA (**2**) could be spontaneously decreased even in a short reaction time (10 min).

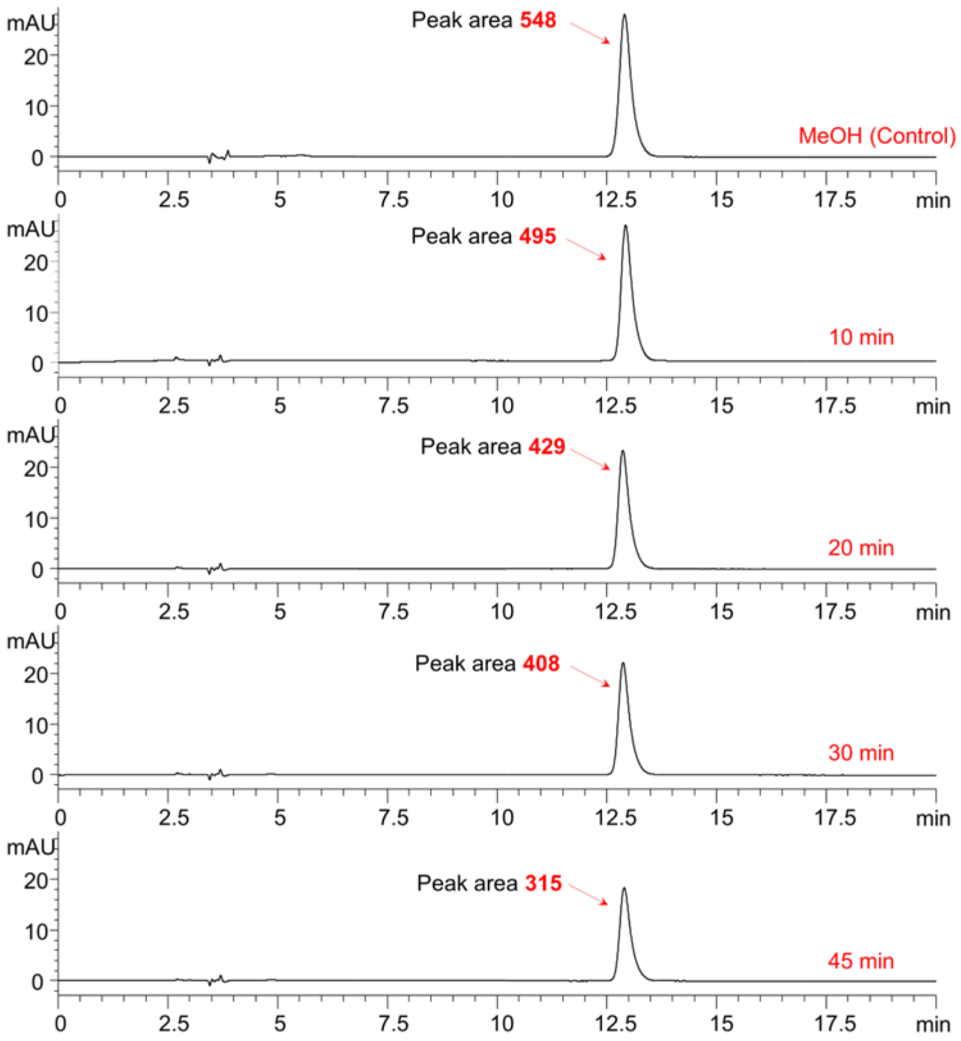

Supplementary Fig. 6. Investigation of the stability of GA (2) at different incubation times. GA (**2**) was added into pH7.0 Na_2HPO_4-NaH_2PO_4 buffer and was incubated for 10, 20, 30, and 45 min, respectively. HPLC analysis results showed that the peak areas of GA (**2**) are 548 (control), 495 (10 min), 429 (20 min), 408 (30min) and 315 (45 min), respectively. These results indicated that GA (**2**) was unstable in phosphate buffer solution.

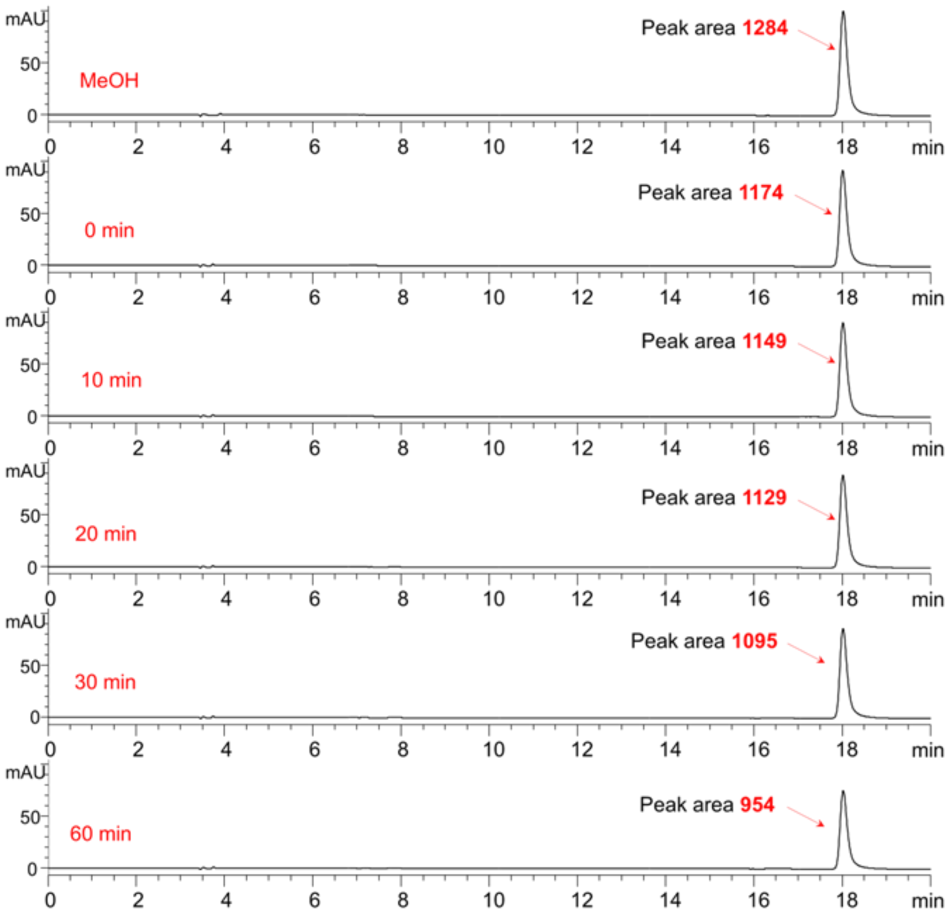

Supplementary Fig. 7. Investigation of the stability of 4-OMGA (3) at different incubation times. 4-OMGA (**3**) was added into pH 8.0 Na_2HPO_4-NaH_2PO_4 buffer and was incubated for 0, 10, 20, 30, and 60 min, respectively. HPLC analysis results showed that the peak areas of 4-OMGA (**3**) are 1284 (control), 1174 (0 min), 1149 (10 min), 1129 (20 min),1095 (30min) and 954 (60 min), respectively. These results indicated that 4-OMGA (**3**) was unstable in phosphate buffer solution.

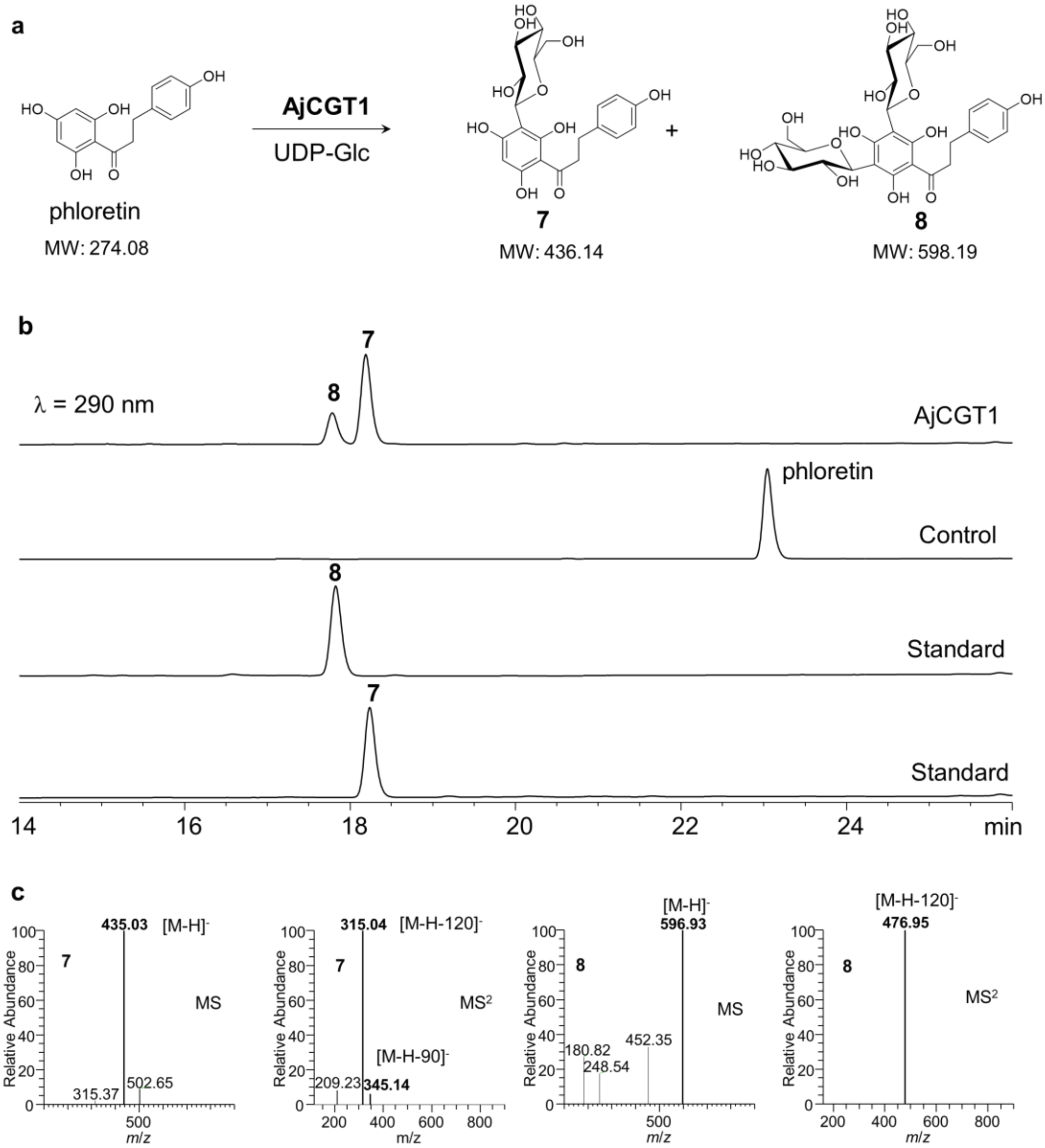

Supplementary Fig. 8. HPLC-MS/MS^2 analysis of AjCGT1 catalyzing the *C*-glycosylation of phloretin. a) AjCGT1 catalyzed the *C*-glycosylation of phloretin; b) HPLC chromatograms of the AjCGT1 catalyzing reaction, control and the standard *C*-glycosides; c) Typical negative ion MS and MS^2 spectra for the *C*-glycosylated products **7** and **8**. The *in vitro* assay of AjCGT1 (in a total volume of 100 µL) consisted of 50 mM Na_2HPO_4-NaH_2PO_4 buffer (pH 8.0), 0.8 mM UDP-Glc, 0.4 mM phloretin and 50 µg purified recombinant AjCGT1, and the mixture was incubated at 40 °C for 6 h.

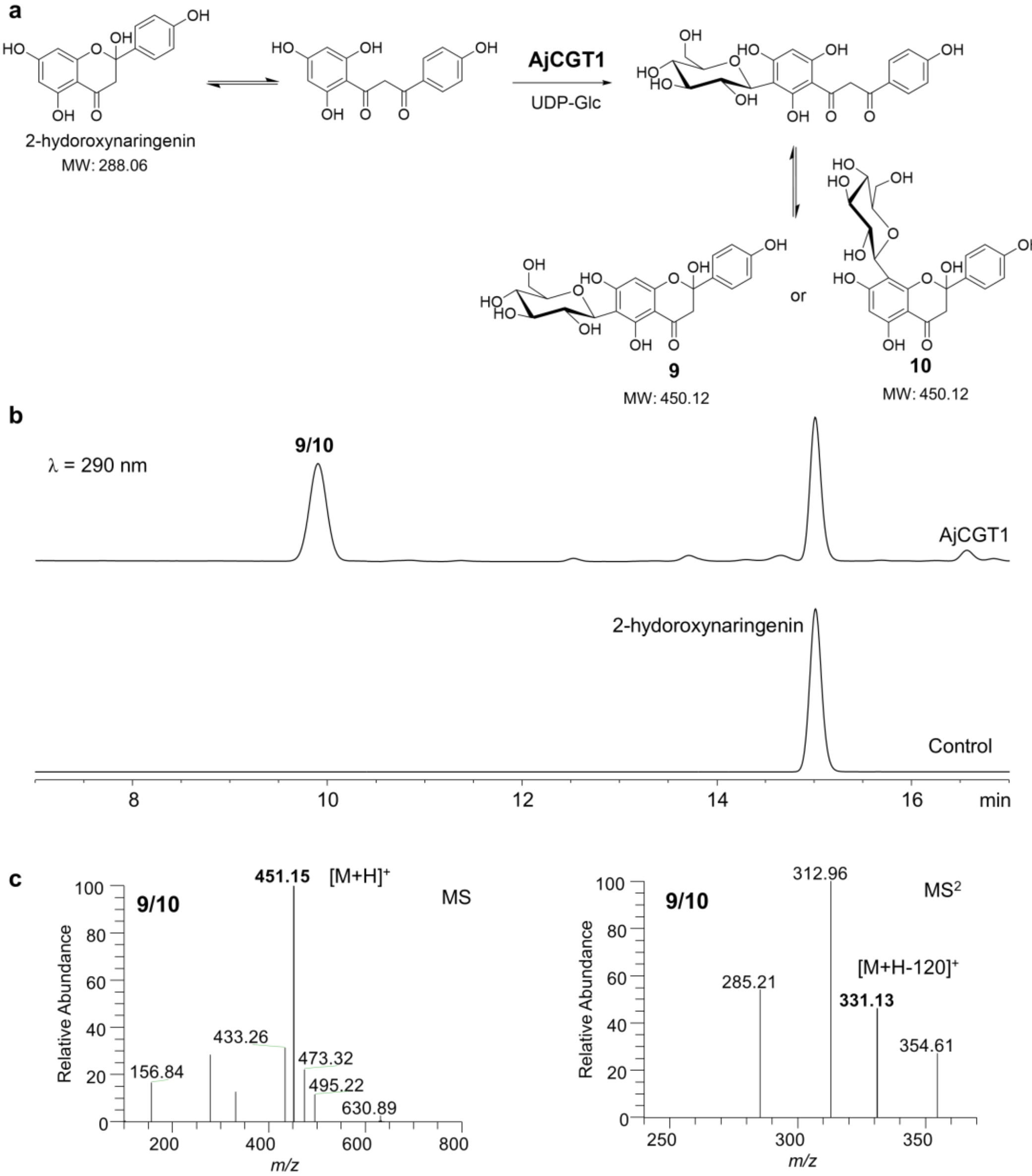

Supplementary Fig. 9. HPLC-MS/MS^2 analysis of AjCGT1 catalyzing the *C*-glycosylation of 2-hydoroxynaringenin. a) AjCGT1 catalyzed the *C*-glycosylation of 2-hydoroxynaringenin; b) HPLC chromatograms of the AjCGT1 catalyzing reaction and the control reaction; c) Typical positive ion MS and MS^2 spectra for the *C*-glycosylated products **9**/**10**. The *in vitro* assay of AjCGT1 (in a total volume of 100 µL) consisted of 50 mM Na_2HPO_4-NaH_2PO_4 buffer (pH 8.0), 0.8 mM UDP-Glc, 0.4 mM 2-hydoroxynaringenin and 50 µg purified recombinant AjCGT1, and the mixture was incubated at 40 °C for 6 h.

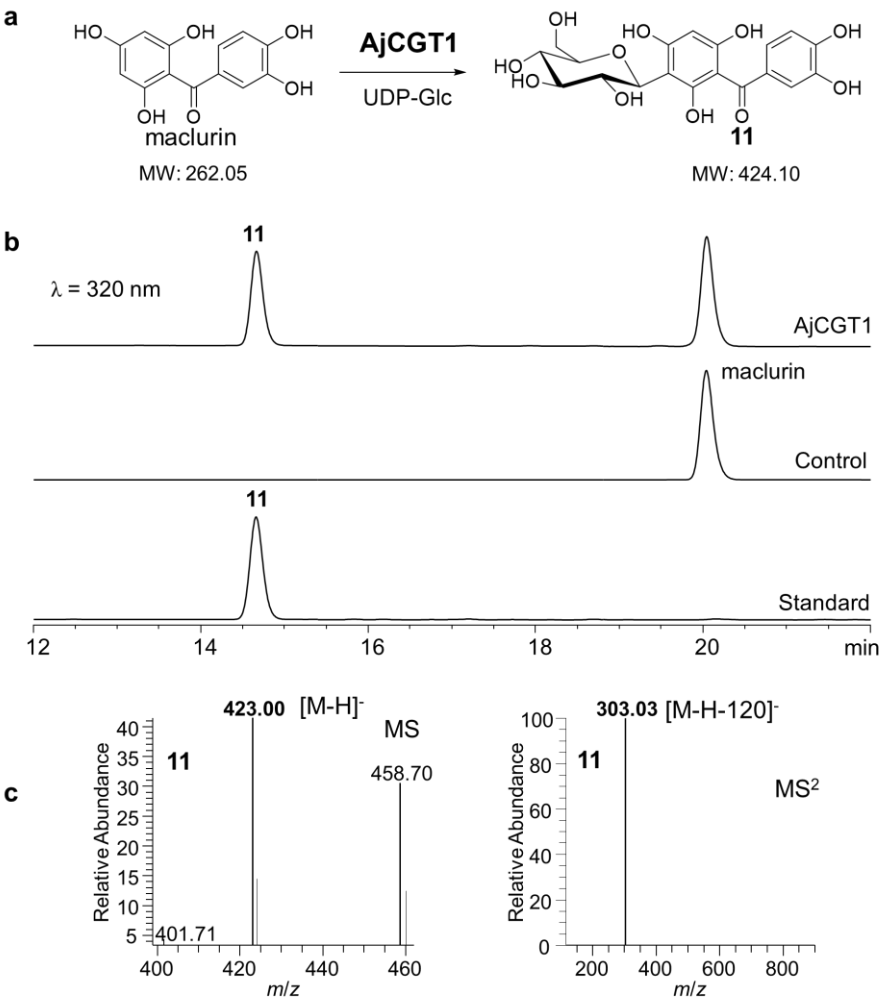

Supplementary Fig. 10. HPLC-MS/MS^2 analysis of AjCGT1 catalyzing the *C*-glycosylation of maclurin. a) AjCGT1 catalyzed the *C*-glycosylation of maclurin; b) HPLC chromatograms of the AjCGT1 catalyzing reaction, control and the standard *C*-glycosides; c) Typical negative ion MS and MS^2 spectra for the *C*-glycosylated product **11**. The *in vitro* assay of AjCGT1 (in a total volume 100 of μL) consisted of 50 mM Na_2HPO_4-NaH_2PO_4 buffer (pH 8.0), 0.8 mM UDP-Glc, 0.4 mM maclurin and 50 μg purified recombinant AjCGT1, and the mixture was incubated at 40 °C for 1 h.

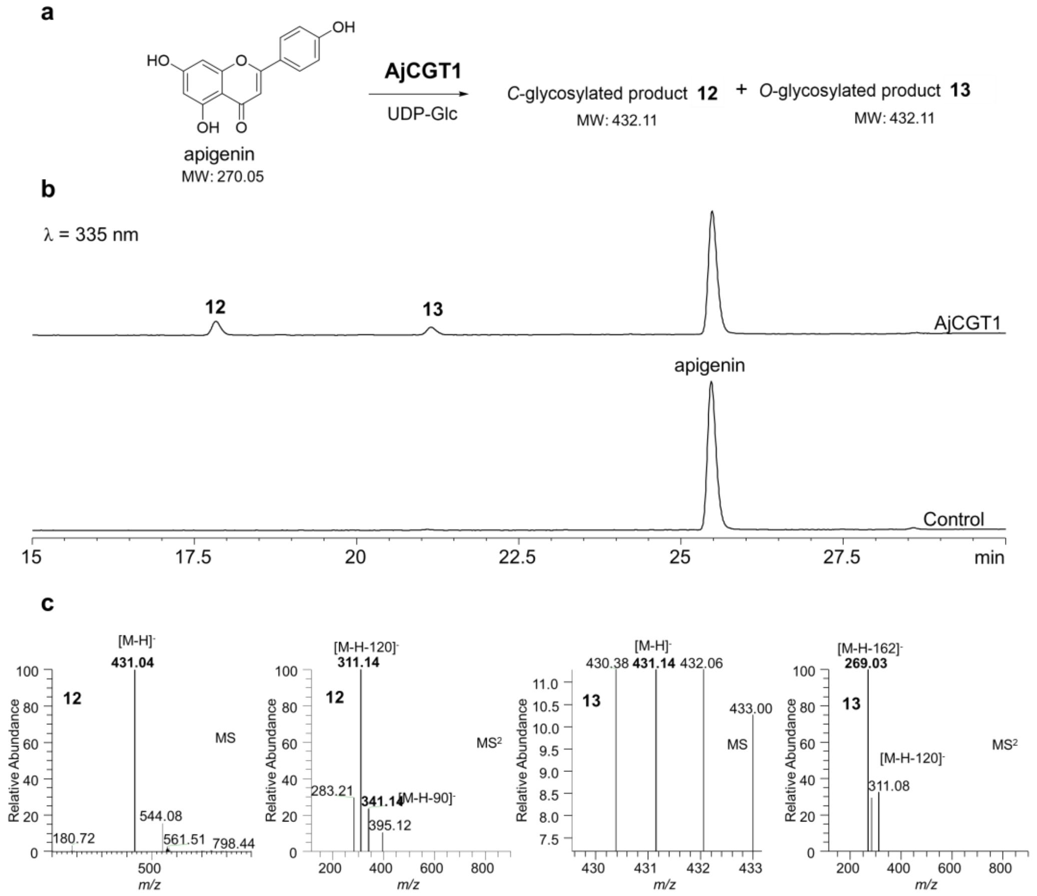

Supplementary Fig. 11. HPLC-MS/MS^2 analysis of AjCGT1 catalyzing the *C*-glycosylation of apigenin. a) AjCGT1 catalyzed the *C*-glycosylation of apigenin; b) HPLC chromatograms of the AjCGT1 catalyzing reaction and the control; c) Typical negative ion MS and MS^2 spectra for the *C*-glycosylated product **12** and *O*-glycosylated product **13**. The *in vitro* assay of AjCGT1 (in a total volume of 100 μL) consisted of 50 mM Na_2HPO_4-NaH_2PO_4 buffer (pH 8.0), 0.8 mM UDP-Glc, 0.4 mM apigenin and 50 μg purified recombinant AjCGT1, and the mixture was incubated at 40 °C for 12 h.

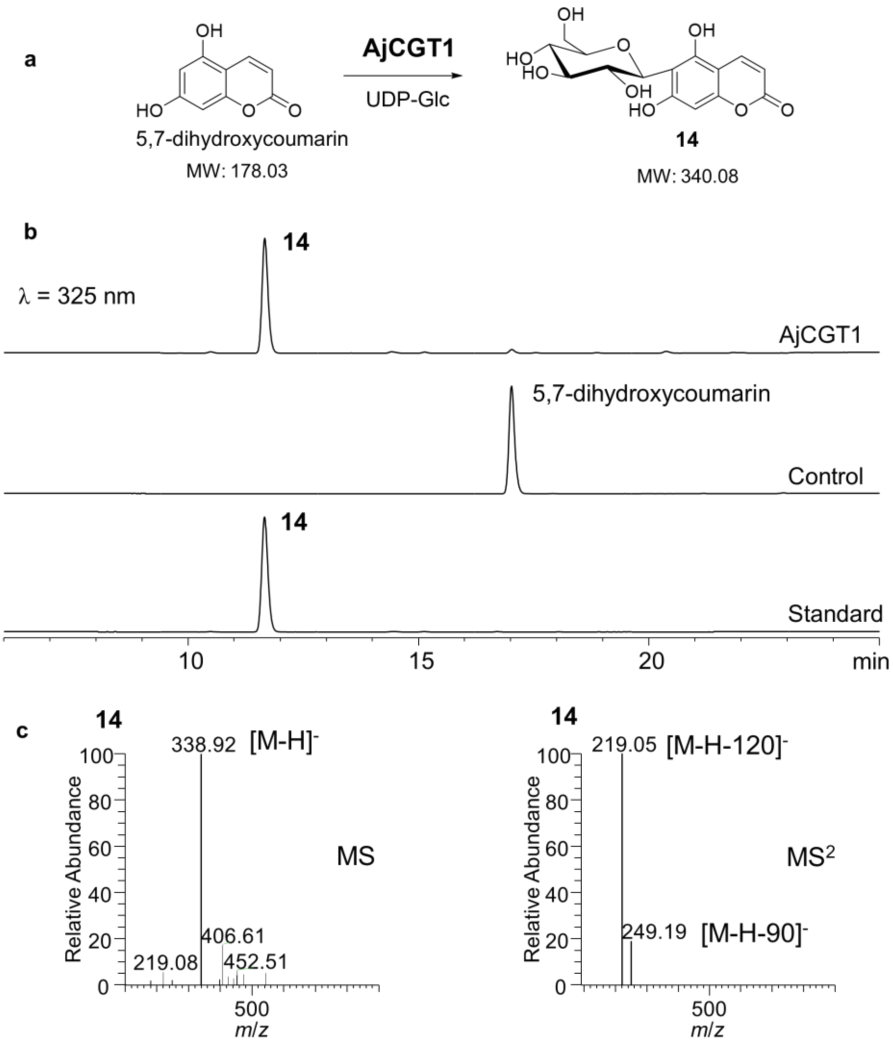

Supplementary Fig. 12. HPLC-MS/MS^2 analysis of AjCGT1 catalyzing the *C*-glycosylation of 5,7-dihydroxycoumarin. a) AjCGT1 catalyzed the *C*-glycosylation of 5,7-dihydroxycoumarin; b) HPLC chromatograms of the AjCGT1 catalyzing reaction, control and the standard *C*-glycoside; c) Typical negative ion MS and MS^2 spectra for the *C*-glycosylated product **14**. The *in vitro* assay of AjCGT1 (in a total volume 100 of µL) consisted of 50 mM Na_2HPO_4-NaH_2PO_4 buffer (pH 8.0), 0.8 mM UDP-Glc, 0.4 mM 5,7-dihydroxycoumarin and 50 µg purified recombinant AjCGT1, and the mixture was incubated at 40 °C for 6 h.

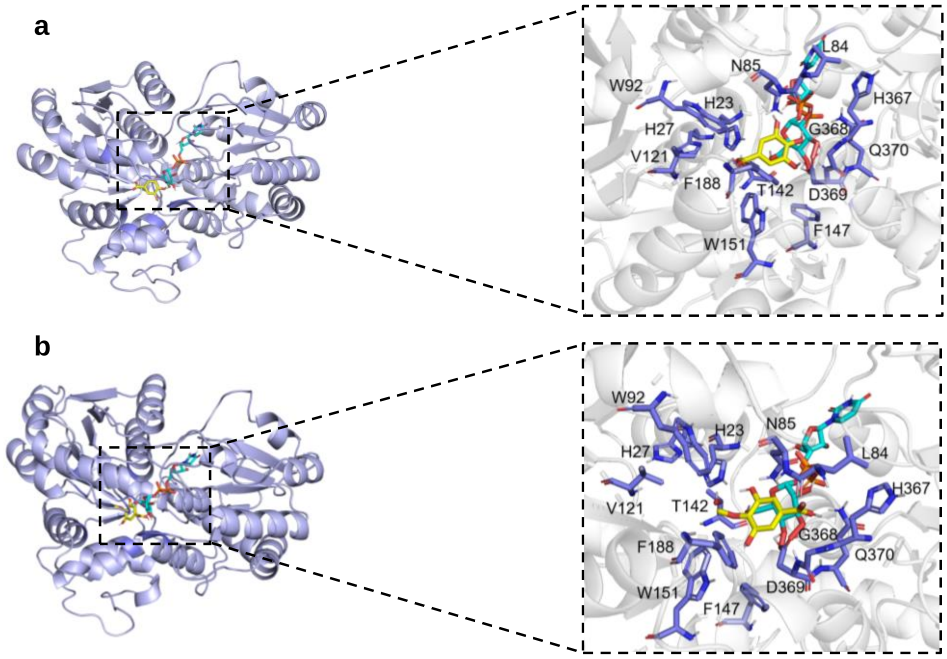

Supplementary Fig. 13. Protein modelling of AjCGT1. The protein was modelled using SbCGTa as a template. GA (**2**) and 4-OMGA (**3**) were docked into the active sites using Autodock Vina. Substrates and UDPG are shown in yellow and green respectively. a) AjCGT1 substrate-binding pockets for GA (**2**), surrounding amino acids hosting GA (**2**) in the active pocket of AjCGT1. b) Substrate binding pockets of AjCGT1 for 4-OMGA (**3**), amino acids surrounding 4-OMGA (**3**) in the active pocket of AjCGT1.

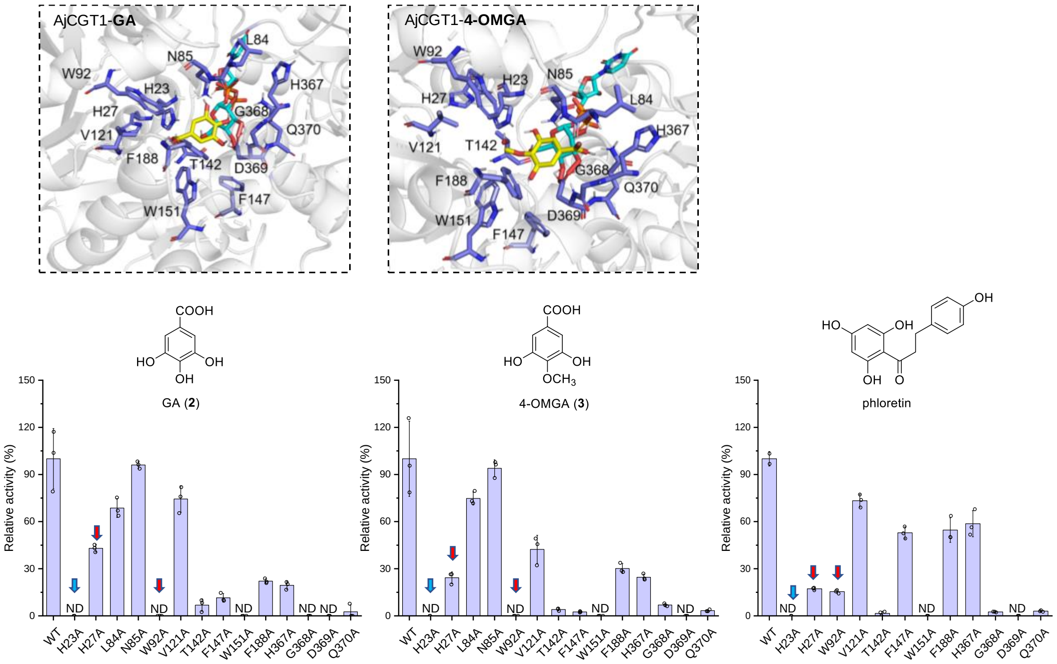

Supplementary Fig. 14. Alanine-scanning of the 14 candidate active sites of AjCGT1. The catalytic activity of the WT and the mutants of AjCGT1 was investigated with GA (**2**), 4-OMGA (**3**) and phloretin as acceptors, respectively (n=3 samples). Bar charts represent mean ± SD of three biologically independent experiments. Source data are provided as a Source Data file.

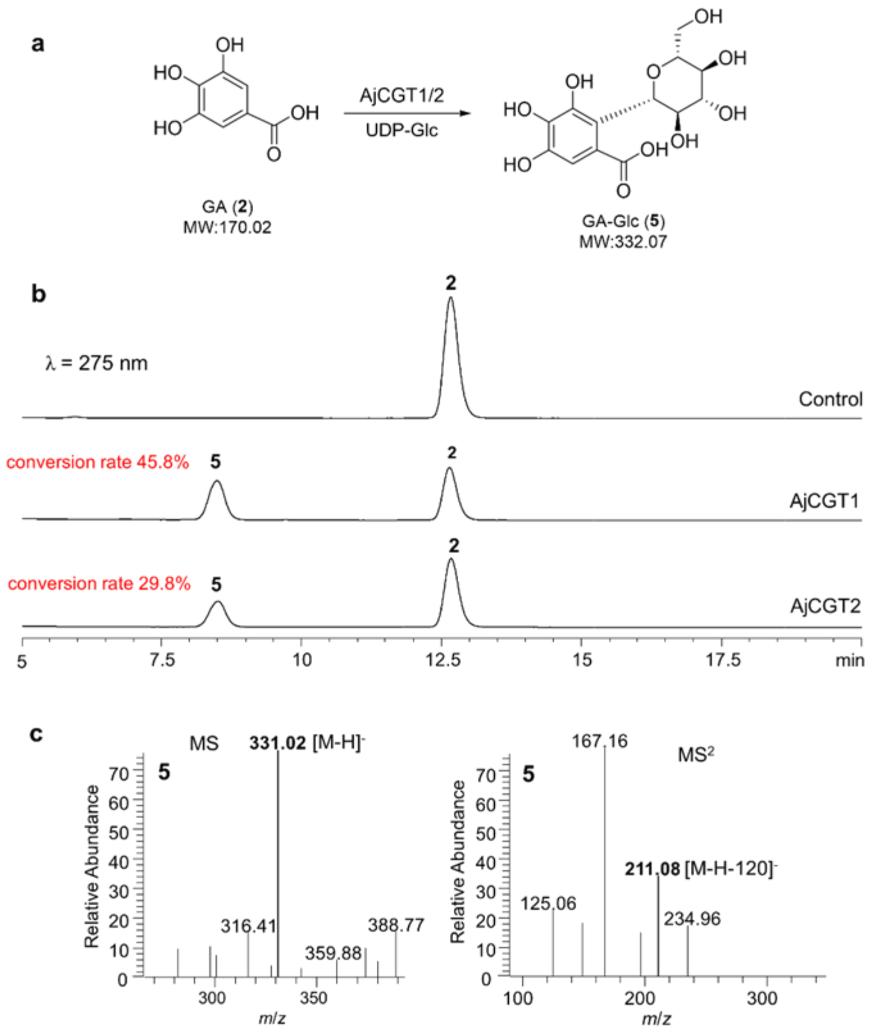

Supplementary Fig. 15. The catalytic activity of AjCGT1 and AjCGT2 with GA (2) as an acceptor. a) AjCGT1 and AjCGT2 catalyzing the *C*-glycosylation of GA (**2**). b) HPLC chromatograms of the AjCGT1 and AjCGT2 catalyzing reactions and control. c) Typical negative ion MS and MS^2 spectra for the *C*-glycosylated product **5**. The *in vitro* assays of CGTs (in a total volume of 100 µL) consisted of 50 mM Tris-HCl buffer (pH 7.0), 0.8 mM UDP-Glc, 0.4 mM GA and 50 µg purified recombinant enzyme, and the mixture was incubated at 40 °C for 1 h.

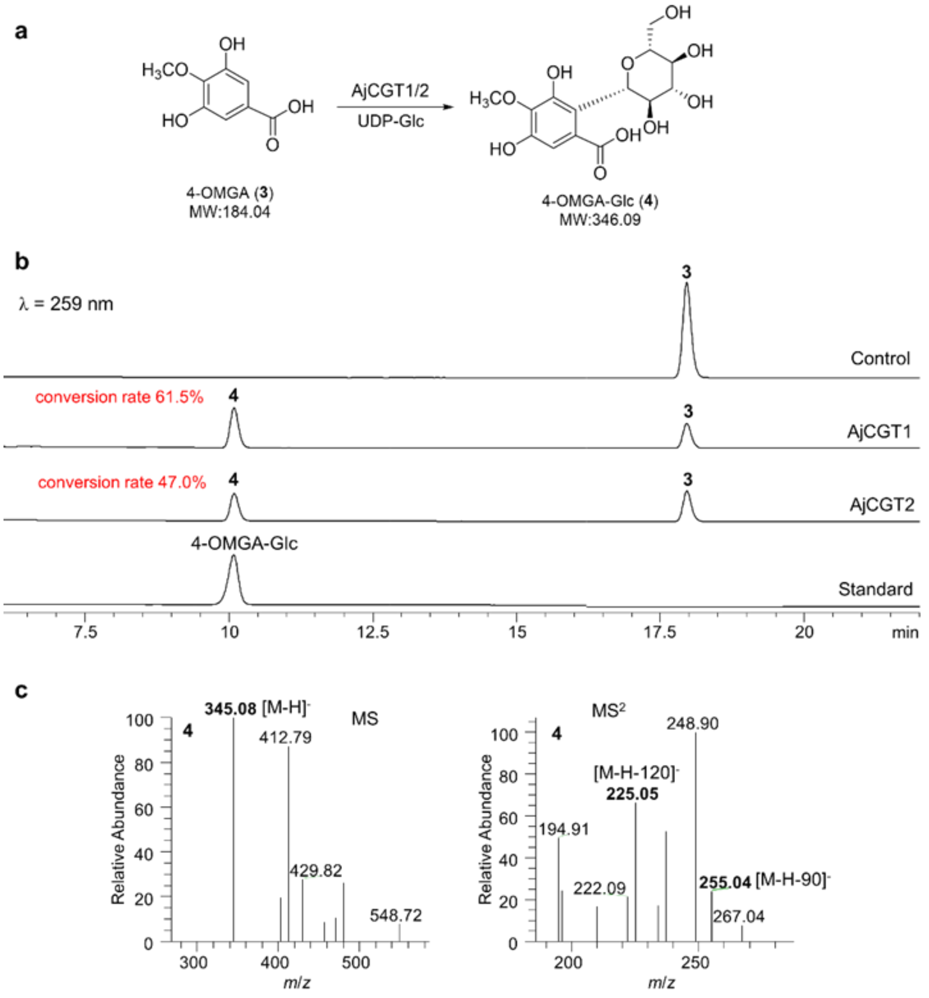

Supplementary Fig. 16. The catalytic activity of AjCGT1 and AjCGT2 with 4-OMGA (3) as an acceptor. a) AjCGT1 and AjCGT2 catalyzing the *C*-glycosylation of 4-OMGA (**3**). b) HPLC chromatograms of the AjCGT1 and AjCGT2 catalyzing reactions, control and the standard 4-OMGA-Glc. c) Typical negative ion MS and MS^2 spectra for the *C*-glycosylated product **4**. The *in vitro* assays of CGTs (in a total volume of 100 μL) consisted of 50 mM Tris-HCl buffer (pH 7.0), 0.8 mM UDP-Glc, 0.4 mM 4-OMGA and 50 μg purified recombinant enzyme, and the mixture was incubated at 40 °C for 1 h.

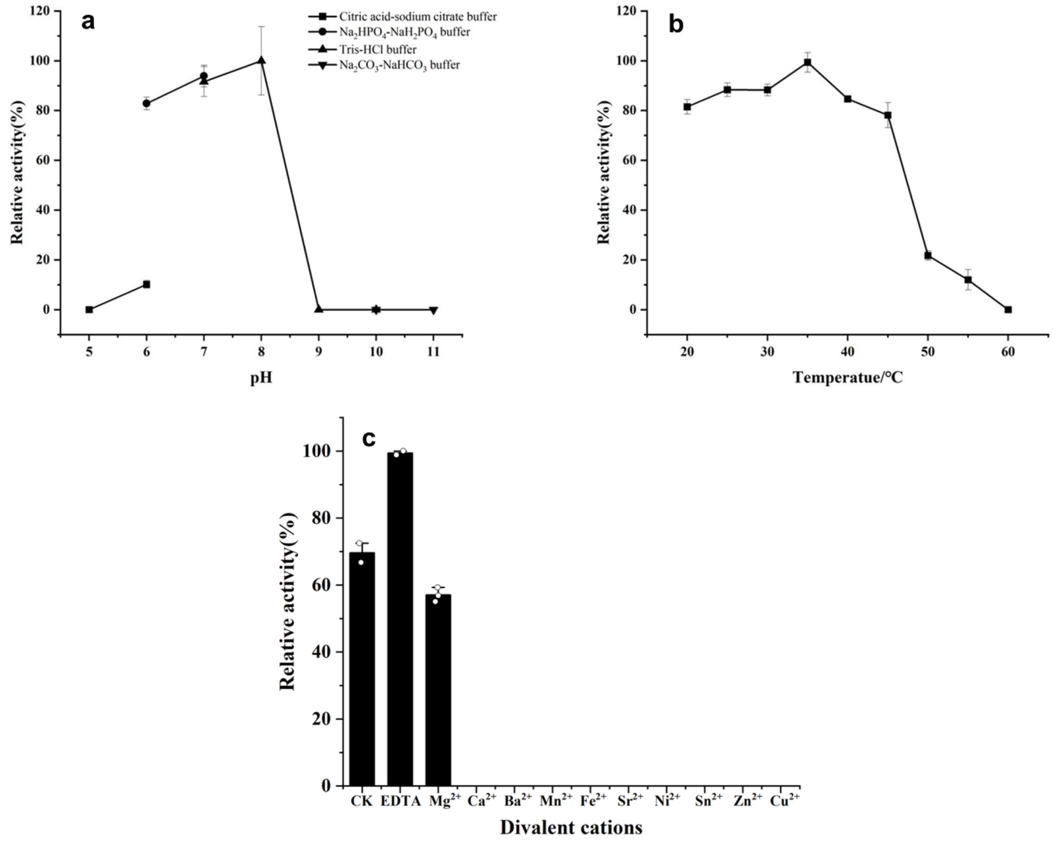

Supplementary Fig. 17. Effects of various pH buffers, temperatures, and divalent metal ions on the activity of AjCGT1 toward GA (2). a) Effects of various pH buffers (pH 5.0–6.0, citric acid-sodium citrate buffer; pH 6.0–7.0, Na_2HPO_4-NaH_2PO_4 buffer; pH 7.0–10.0, Tris-HCl buffer; pH 10.0–11.0, Na_2CO_3-$NaHCO_3$ buffer) (n=3 samples); b) Effects of various temperatures (n=3 samples); c) Effects of various divalent metal ions. GA (**2**) was used as the acceptor (n=3 samples). Bar charts and line charts represent mean ± SD of three biologically independent experiments. Source data are provided as a Source Data file.

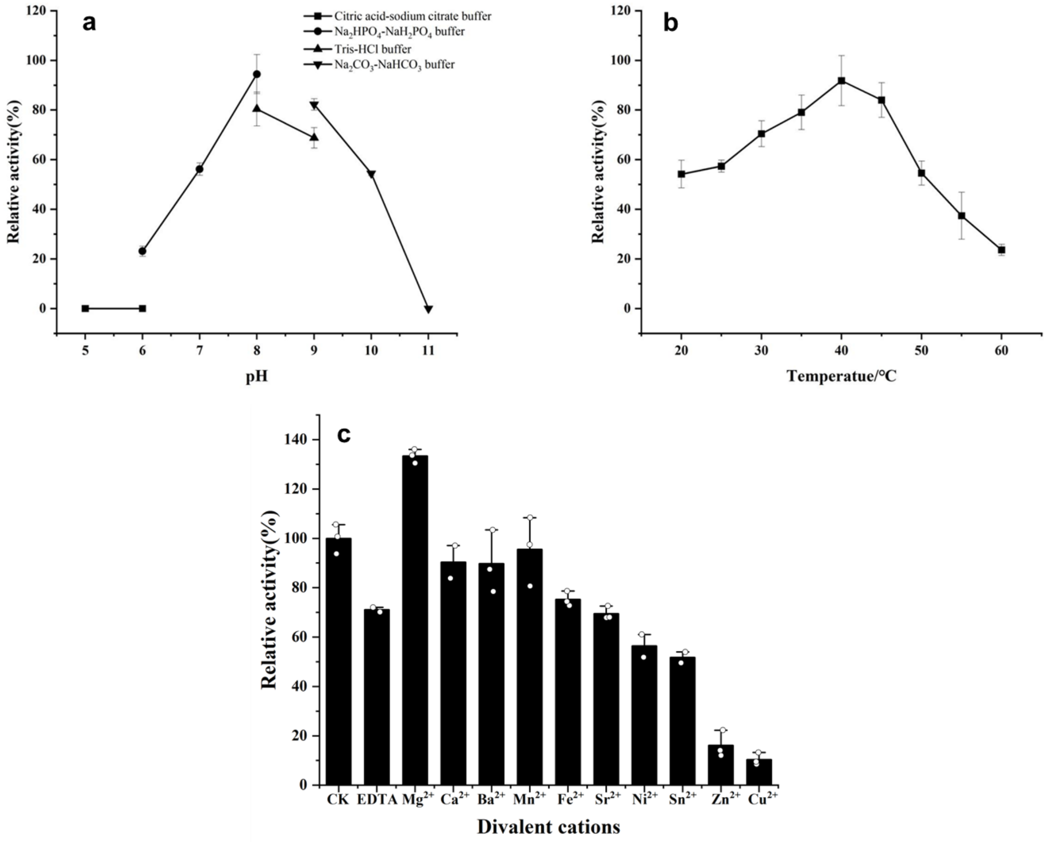

Supplementary Fig. 18. Effects of various pH buffers, temperatures, and divalent metal ions on the activity of AjCGT1 toward 4-OMGA (3). a) Effects of various pH buffers (pH 5.0–6.0, citric acid-sodium citrate buffer; pH 6.0–8.0, Na_2HPO_4-NaH_2PO_4 buffer; pH 8.0–9.0, Tris-HCl buffer; pH 9.0–11.0, Na_2CO_3-$NaHCO_3$ buffer) (n=3 samples); b) Effects of various temperatures (n=3 samples); c) Effects of various divalent metal ions. 4-OMGA (**3**) was used as the acceptor (n=3 samples). Bar charts and line charts represent mean ± SD of three biologically independent experiments. Source data are provided as a Source Data file.

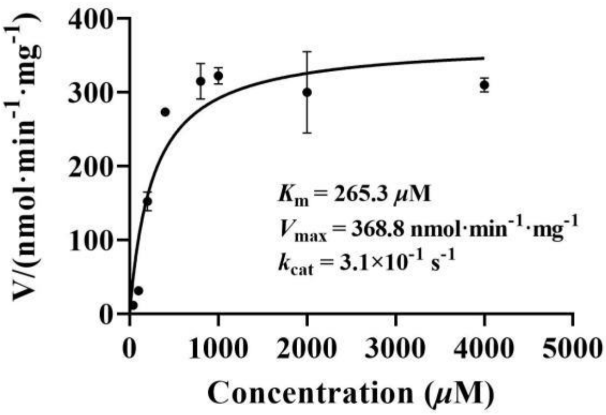

Supplementary Fig. 19. Determination of kinetic parameters for recombinant AjCGT1 with the substrate of 4-OMGA (3). UDP-Glc was used as the sugar donor. The reactions were performed at 40 °C and pH 8.0 (Na_2HPO_4-NaH_2PO_4 buffer) with a short reaction time (1 min) and an increased amount of enzyme (50 μg) in a total volume of 100 μL to avoid the effect of the instability of 4-OMGA (**3**) (n=3 samples).Charts represent mean ± SD of three biologically independent experiments. Source data are provided as a Source Data file.

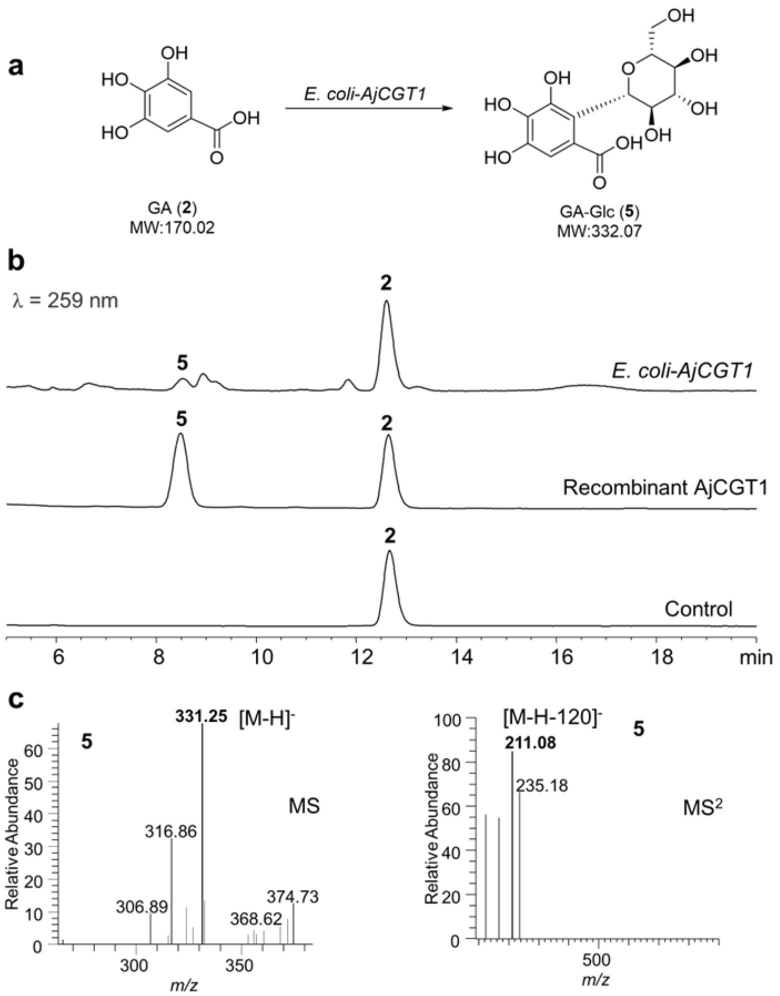

Supplementary Fig. 20. The transformation of GA (2) into GA-Glc (5) by *E. coli-AjCGT1*. a) *E. coli-AjCGT1* catalyzed the *C*-glycosylation of GA (**2,** final concentration 0.4 mM). b) HPLC analysis of the whole cell catalytic product of *E. coli-AjCGT1* and the reaction catalyzed by AjCGT1 *in vitro*. c) Typical negative ion MS and MS^2 spectra for transformed product **5**.

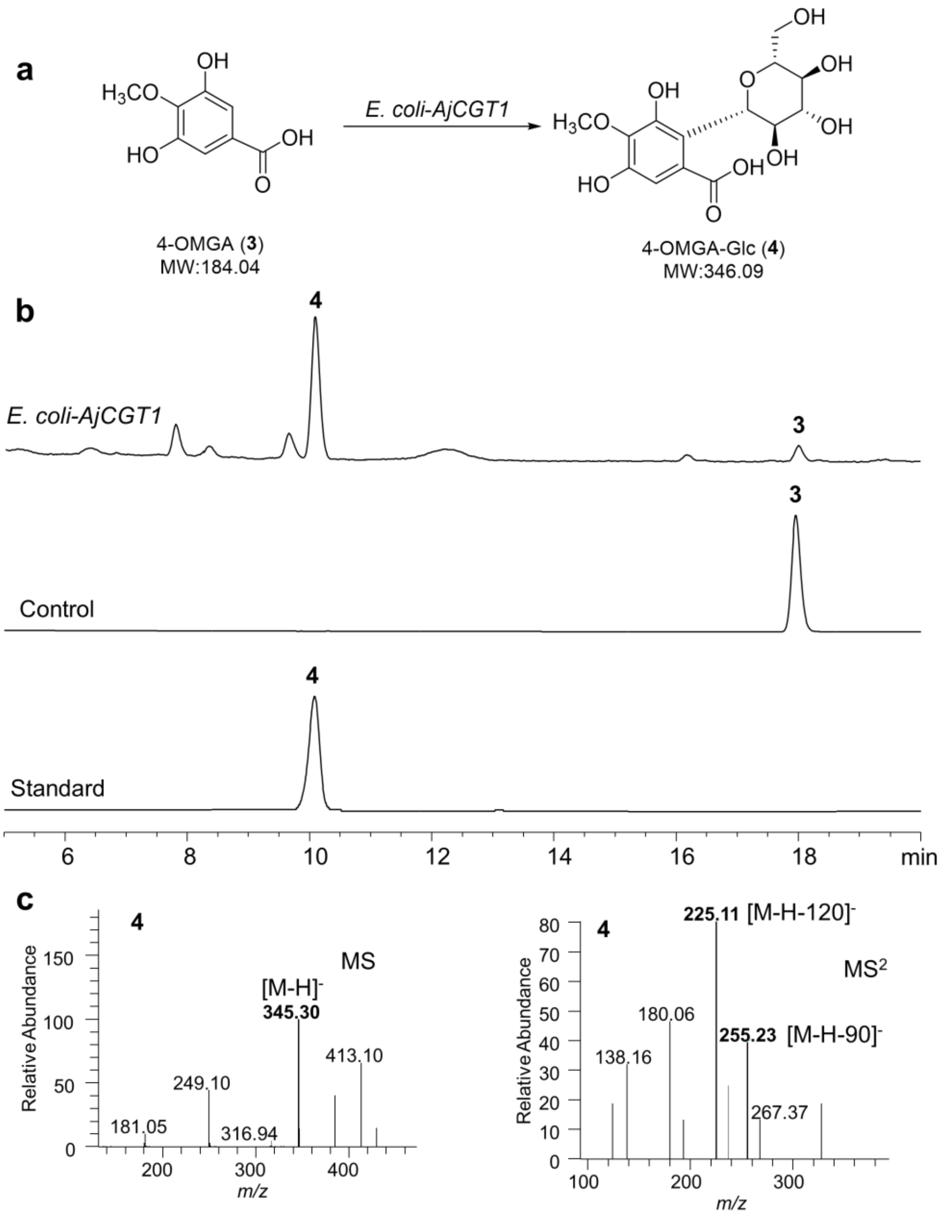

Supplementary Fig. 21. The transformation of 4-OMGA (3) into 4-OMGA-Glc (4) by *E. coli-AjCGT1*. a) *E. coli-AjCGT1* catalyzed the *C*-glycosylation of 4-OMGA (**3**, final concentration 0.4 mM). b) HPLC analysis of the whole cell catalytic product of *E. coli-AjCGT1*, the control reaction and the standard. c) Typical negative ion MS and MS^2 spectra for transformed product **4**.

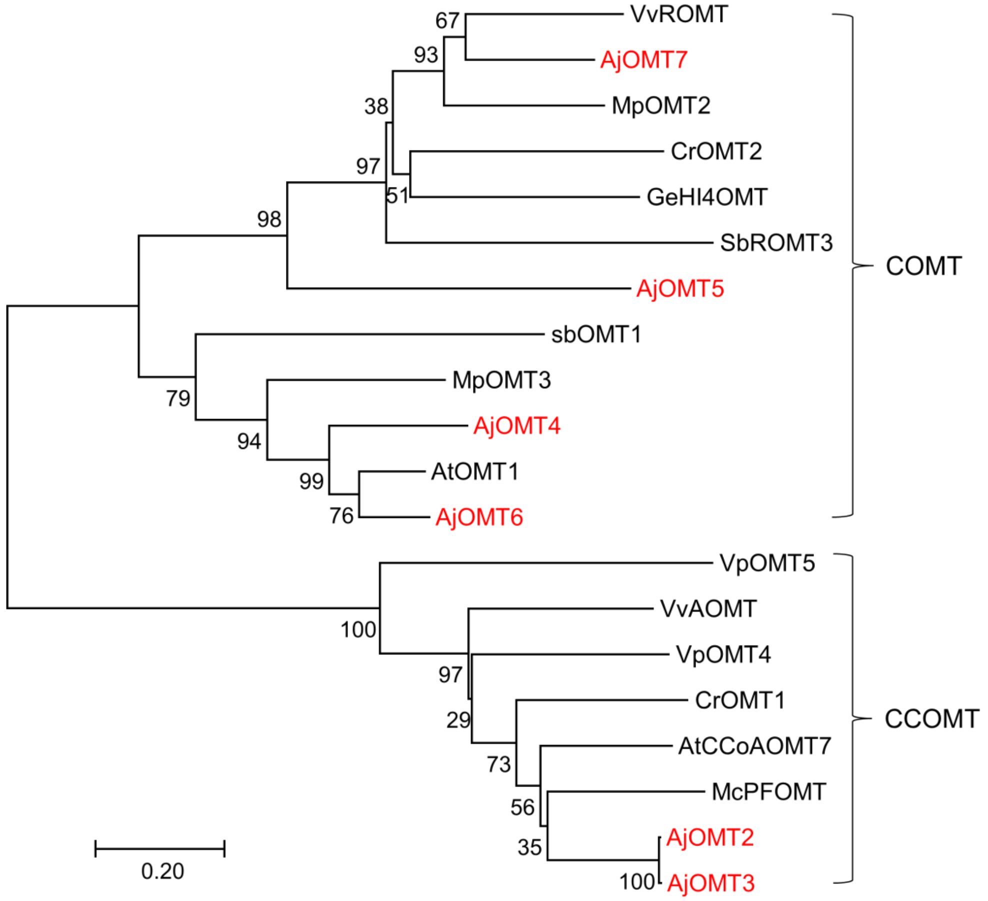

Supplementary Fig. 22. Phylogenetic analysis of the six AjOMTs (in red) with the reported OMTs. COMT: caffeic acid *O*-methyltransferase; CCOMT: caffeoyl-CoA *O*-methyltransferase. The neighbor-joining phylogenetic tree was inferred using the software MEGA 7.0. The bootstrap values are 1000 replicates, and the scale bar represents 0.2 amino acid substitutions per site. The GeneBank accession numbers of OMTs used for analysis are as follows: AtCCoAOMT7 (AEE85172); AtOMT1 (U70424); CrOMT1 (KI536925); CrOMT2 (AY127568); McPFOMT (3C3Y_A); MpOMT2 (AY337459); MpOMT3 (AY337460); GeHI4OMT (AB091684); SbOMT1 (EF189707); SbROMT3 (JX673942); VpOMT4 (JF344740); VpOMT5 (JF344741); VvAOMT (FJ460168); VvROMT (NM_001281115).

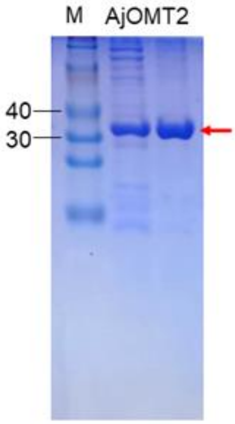

Supplementary Fig. 23. SDS-PAGE of recombinant His$_6$-AjOMT2 purified by affinity chromatography. Lane M: Protein Marker; Lane AjOMT2: His-tagged AjOMT2 (indicated with an arrow, predicted M.W., 31.3 kDa) purified on Ni Sepharose column chromatography. Source data are provided as a Source Data file.

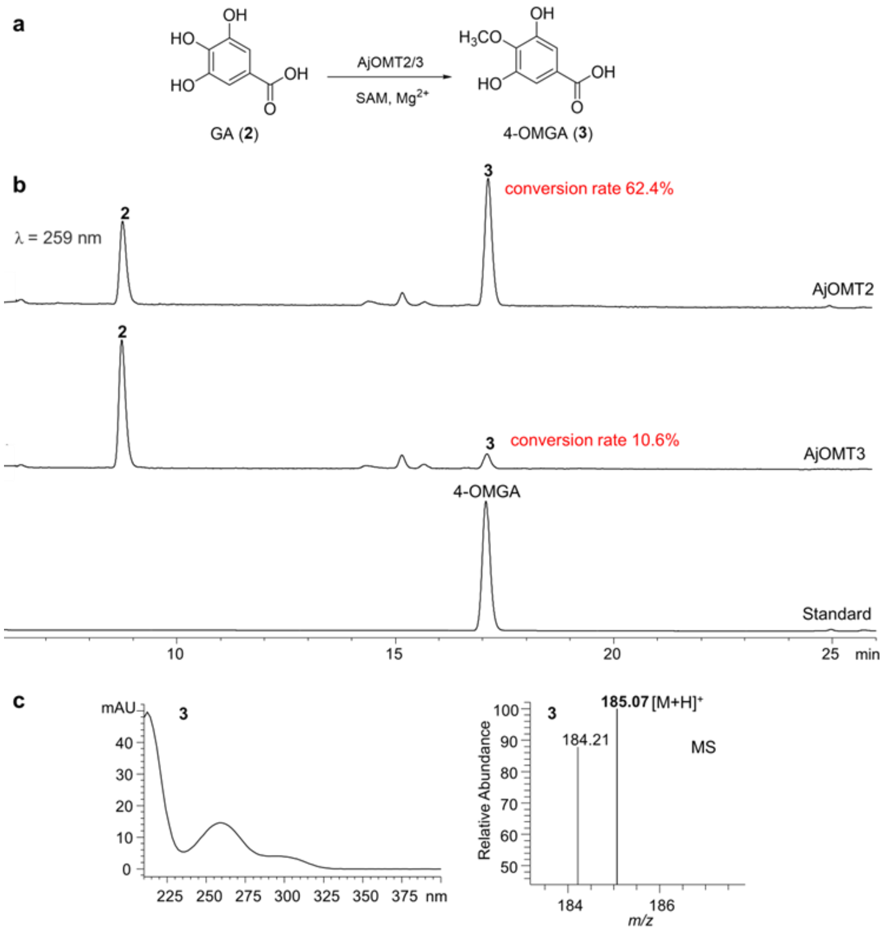

Supplementary Fig. 24. The catalytic activity of AjOMT2 and AjOMT3 with GA (2) as an acceptor. a) AjOMT2 and AjOMT3 catalyzing the *O*-methylation of GA (**2**). b) HPLC chromatograms of the AjOMT2 and AjOMT3 catalyzing reactions and the standard 4-OMGA. c) UV absorption spectrum and typical positive ion MS spectra for the product **3**. The detailed reaction conditions are shown in Methods.

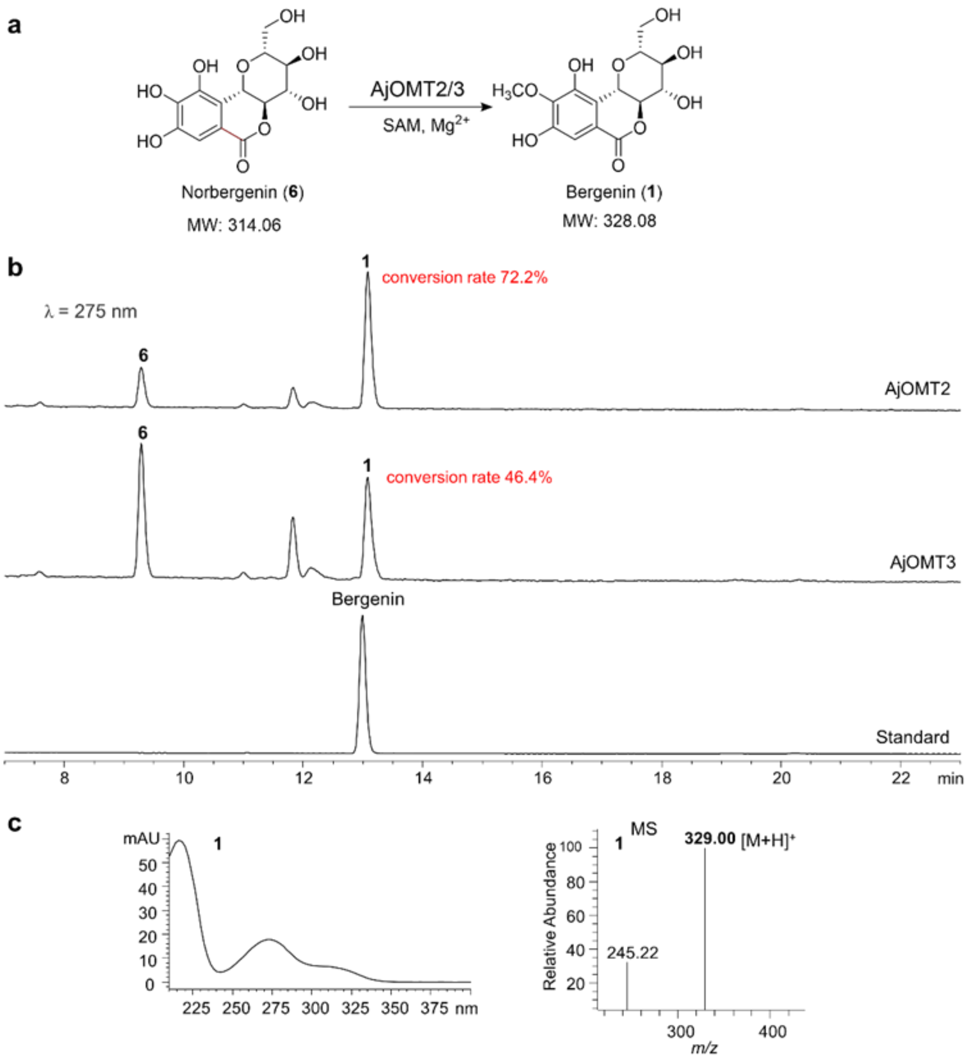

Supplementary Fig. 25. The catalytic activity of AjOMT2 and AjOMT3 with norbergenin (6) as an acceptor. a) AjOMT2 and AjOMT3 catalyzing the *O*-methylation of norbergenin (**6**). b) HPLC chromatograms of the AjOMT2 and AjOMT3 catalyzing reactions and the standard bergenin. c) UV absorption spectrum and typical positive ion MS spectra for the product **1**. The detailed reaction conditions are shown in Methods.

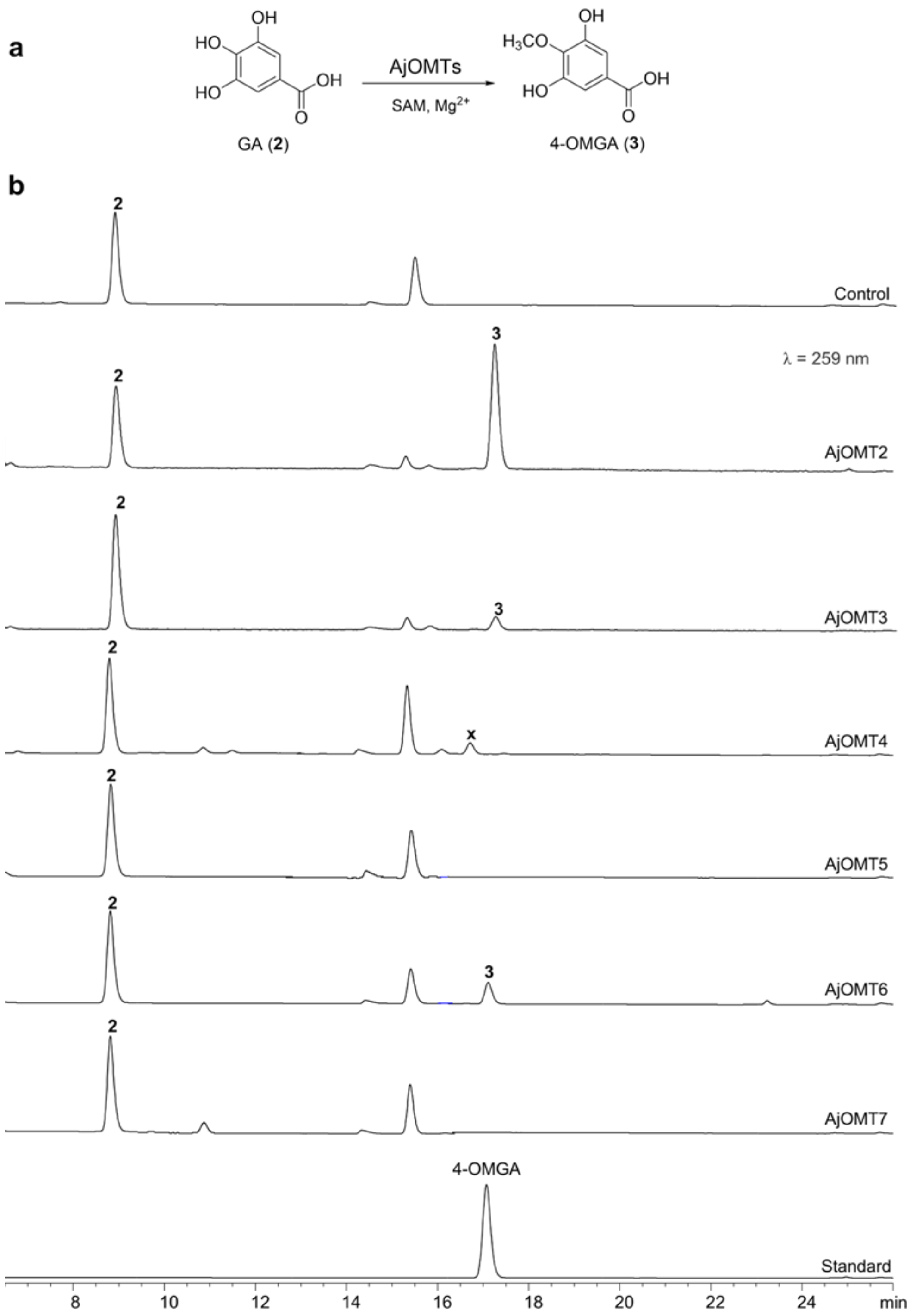

Supplementary Fig. 26. The catalytic *O*-methylation activity of AjOMTs towards GA (2). a) *O*-methylation of GA (**2**) reactions catalyzed by AjOMTs; b) HPLC analysis of the enzymatic reactions. The detailed reaction conditions are shown in Methods.

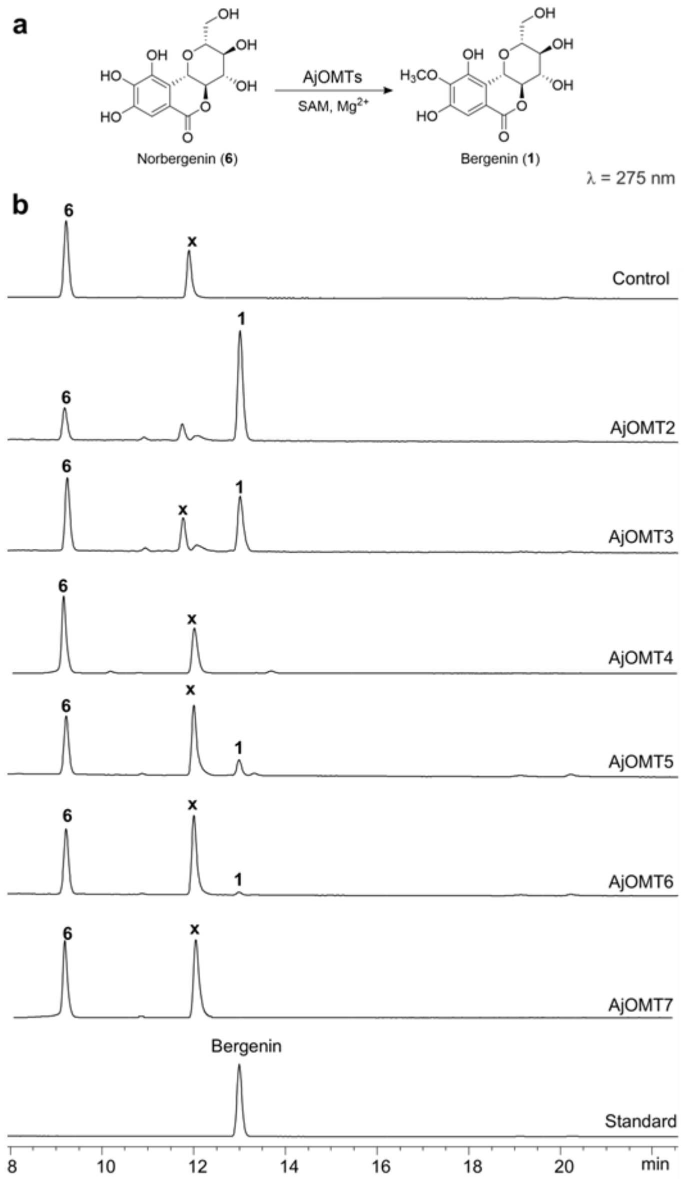

Supplementary Fig. 27. The catalytic *O*-methylation activity of AjOMTs towards norbergenin (6). a) *O*-methylation of norbergenin (**6**) reactions catalyzed by AjOMTs; b) HPLC analysis of the enzymatic reactions. The detailed reaction conditions are shown in Methods.

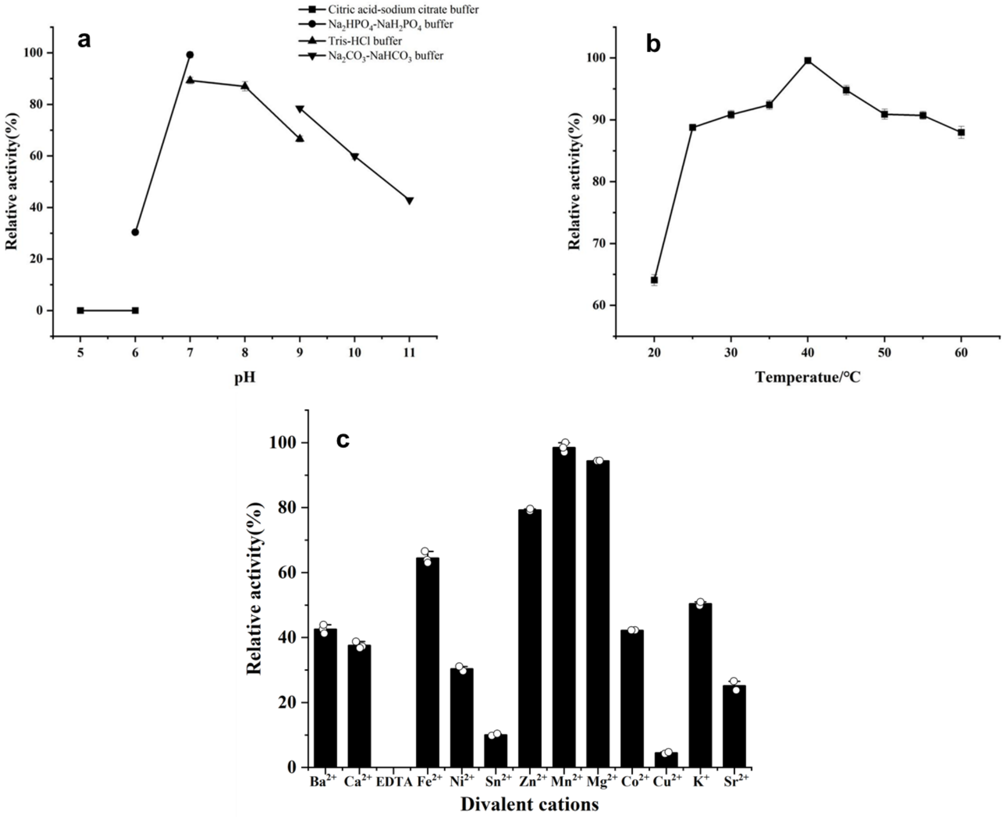

Supplementary Fig. 28. Effects of various pH buffers, temperatures, and divalent metal ions on the activity of AjOMT2 toward GA (2). a) Effects of various pH buffers (pH 5.0–6.0, citric acid-sodium citrate buffer; pH 6.0–7.0, Na_2HPO_4-NaH_2PO_4 buffer; pH 7.0–9.0, Tris-HCl buffer; pH 9.0–11.0, Na_2CO_3-$NaHCO_3$ buffer) (n=3 samples); b) Effects of various temperatures (n=3 samples); c) Effects of various divalent metal ions. SAM was used as a methyl donor and GA (**2**) was used as acceptor (n=3 samples). Bar charts and line charts represent mean ± SD of three biologically independent experiments. Source data are provided as a Source Data file.

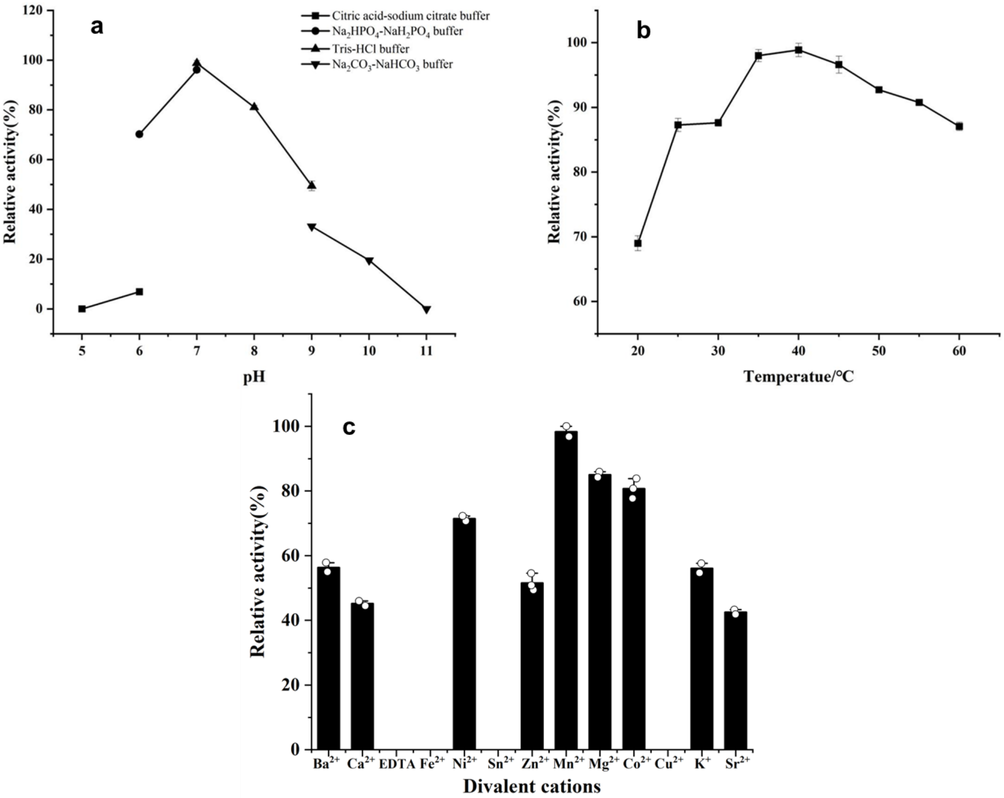

Supplementary Fig. 29. Effects of various pH buffers, temperatures, and divalent metal ions on the activity of AjOMT2 toward norbergenin (6). a) Effects of various pH buffers (pH 5.0–6.0, citric acid-sodium citrate buffer; pH 6.0–7.0, Na_2HPO_4-NaH_2PO_4 buffer; pH 7.0–9.0, Tris-HCl buffer; pH 9.0–11.0, Na_2CO_3-$NaHCO_3$ buffer) (n=3 samples); b) Effects of various temperatures (n=3 samples); c) Effects of various divalent metal ions. SAM was used as a methyl donor and norbergenin (**6**) was used as acceptor (n=3 samples).Bar charts and line charts represent mean ± SD of three biologically independent experiments. Source data are provided as a Source Data file.

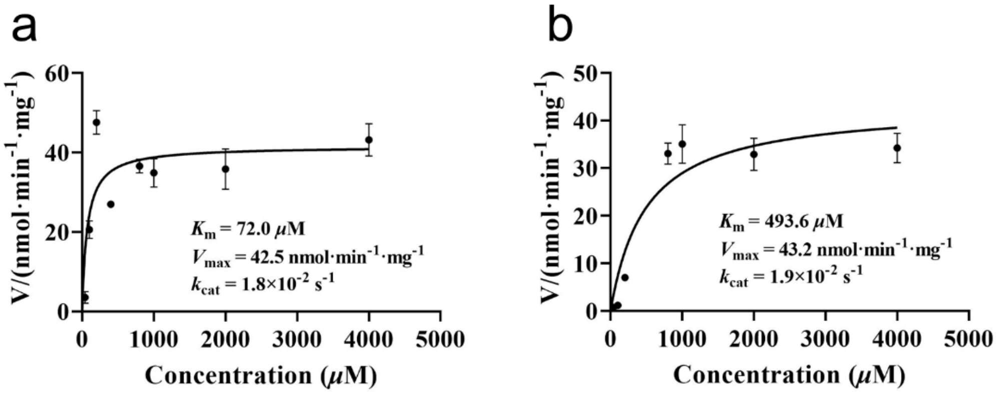

Supplementary Fig. 30. Determination of kinetic parameters for recombinant AjOMT2. Kinetic parameters were detected with gallic acid (**a**) and norbergenin (**b**) as acceptors and SAM as a methyl donor (n=3 samples).Charts represent mean ± SD of three biologically independent experiments. Source data are provided as a Source Data file.

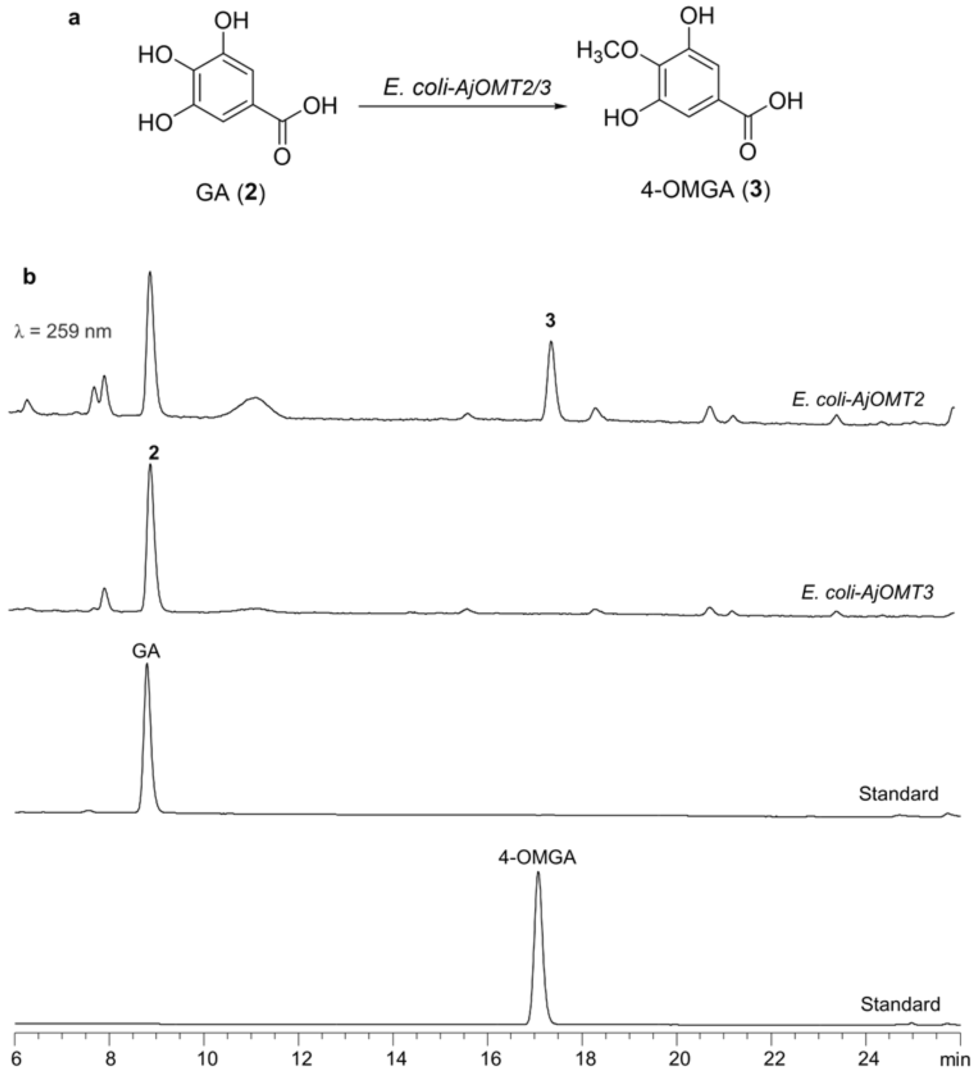

Supplementary Fig. 31. The transformation of GA (2) into 4-OMGA (3) by *E. coli-AjOMT2*/3.
a) *E. coli-AjOMT2/3* catalyzed the *O*-methylation of GA (**2,** final concentration 0.4 mM). b) HPLC analysis of the whole cell catalytic product of *E. coli-AjOMT2/3* and the standards.

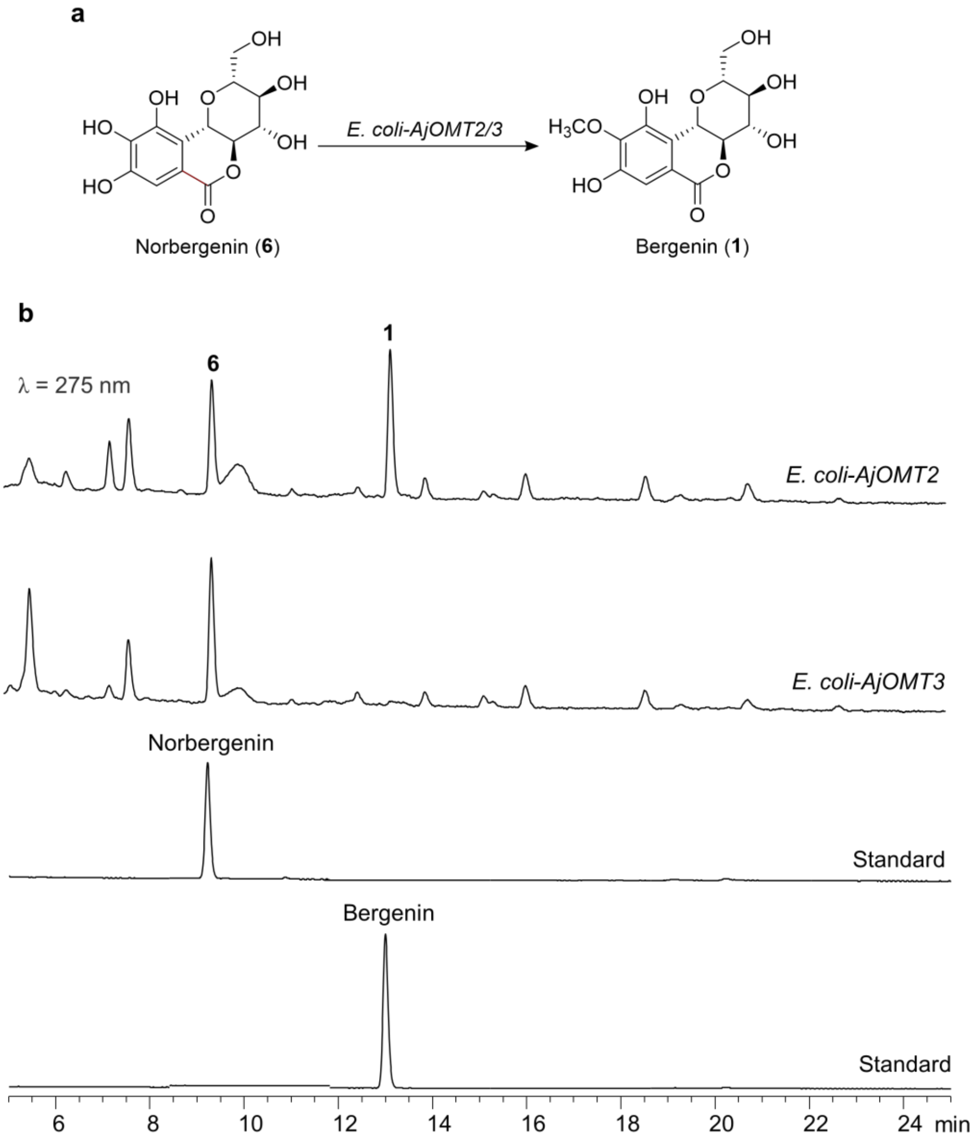

Supplementary Fig. 32. The transformation of norbergenin (6) into bergenin (1) by *E. coli-AjOMT2*/3. a) *E. coli-AjOMT2/3* catalyzed the *O*-methylation of norbergenin (**6,** final concentration 0.4 mM). b) HPLC analysis of the whole cell catalytic product of *E. coli-AjOMT2/3* and the standards.

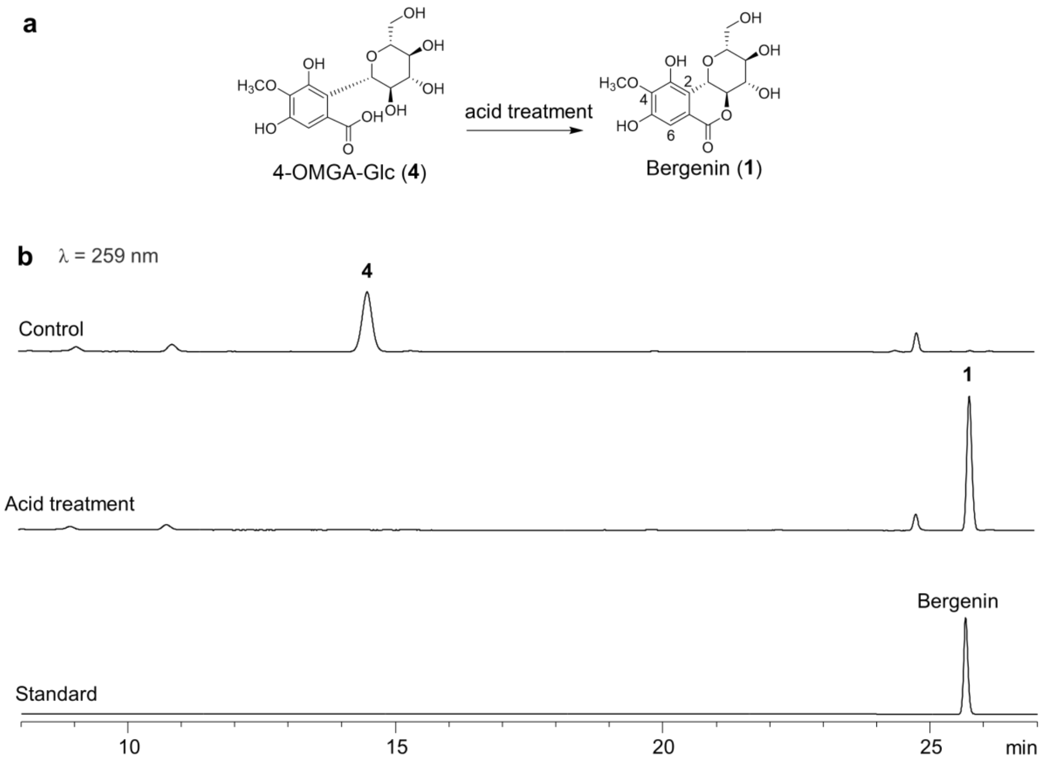

Supplementary Fig. 33. The conversion of 4-OMGA-Glc (4) to bergenin (1) by acid treatment.
a) The transforming reaction of 4-OMGA-Glc (**4**); b) HPLC analysis of the transforming reaction.

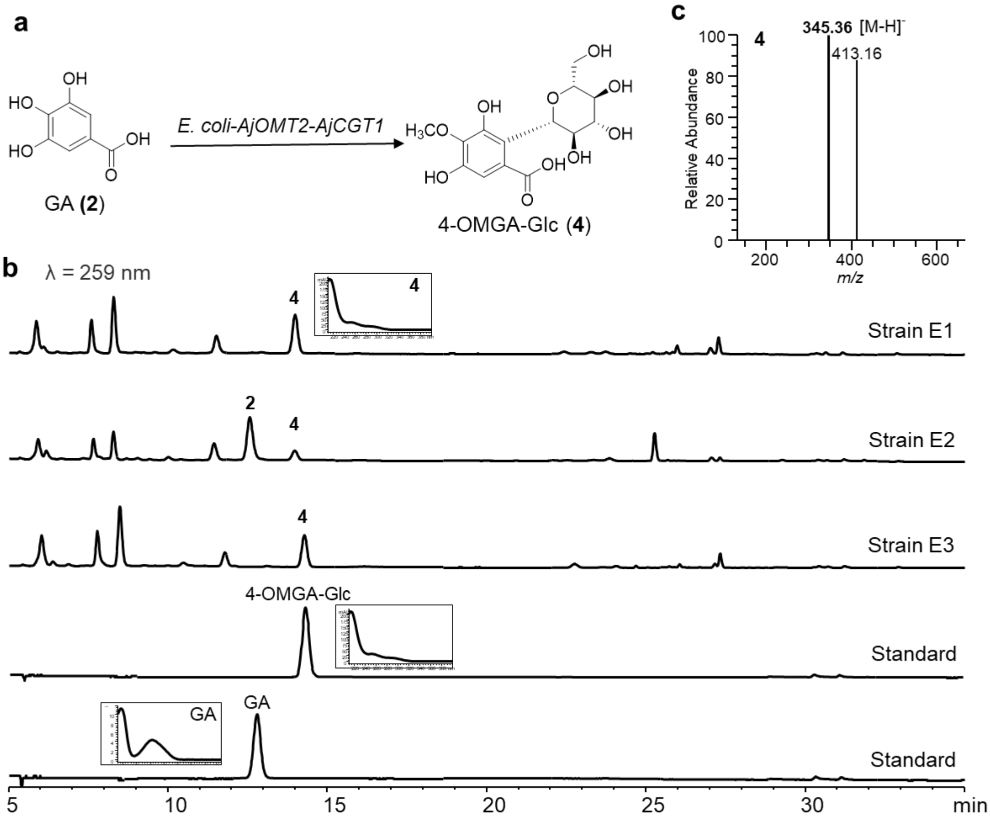

Supplementary Fig. 34. GA (2) was transformed to 4-OMGA-Glc (4) by *E. coli-AjOMT2-AjCGT1*. a) *E. coli-AjOMT2-AjCGT1* catalyzed the *O*-methylation and *C*-glycosylation of GA (**2**). b) HPLC analysis of the whole cell catalytic product of different strains. c) Typical negative-ion MS spectrum for **4**. The final concentration of GA (**2**) in the reactions is 0.4 mM.

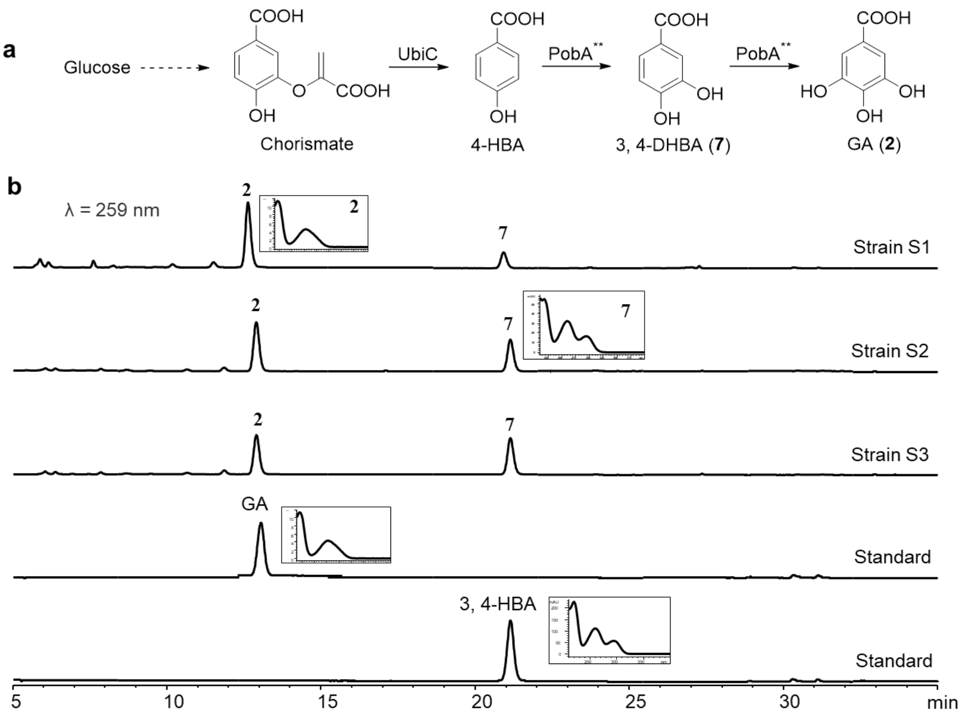

Supplementary Fig. 35. *De novo* synthesis of GA (2) by engineered strains S1−S3. a) The artificial pathway to GA (**2**) from glucose constructed in *E. coli*; b) HPLC-UV analysis of the culture supernatant of the fermented recombinant strains S1−S3, respectively.

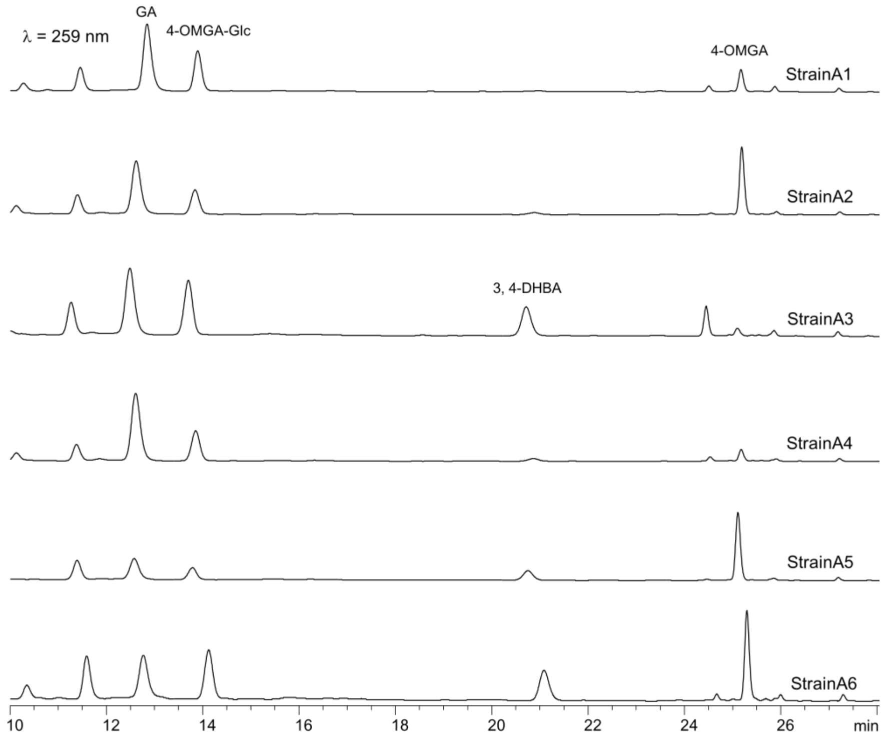

Supplementary Fig. 36. The fermentation broth supernatants of strains A1−A6 were analyzed by HPLC after inducing expression for 24 h.

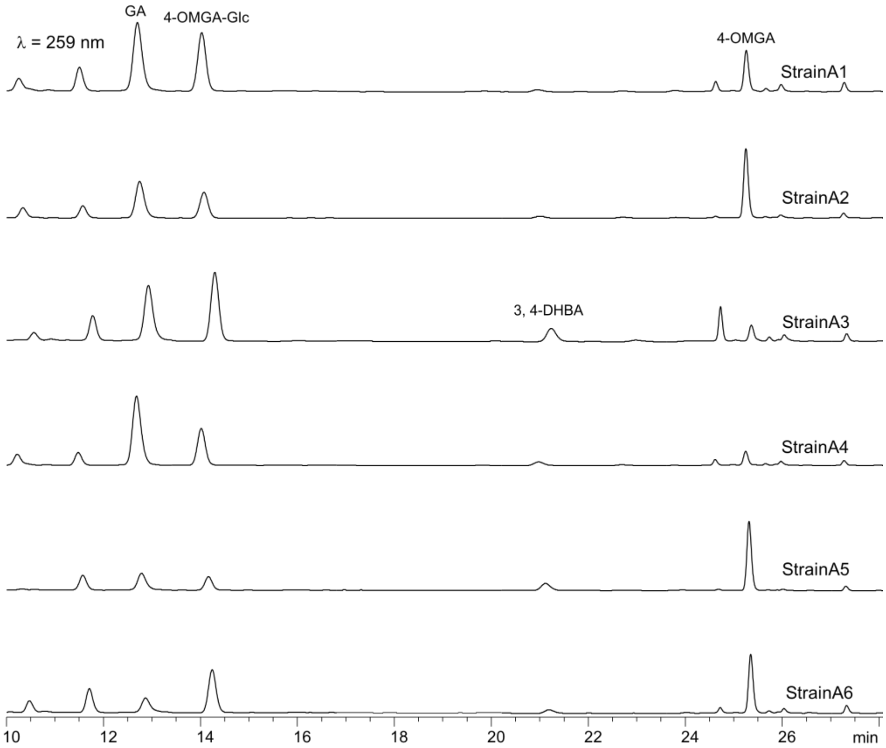

Supplementary Fig. 37. The fermentation broth supernatants of strains A1–A6 were analyzed by HPLC after inducing expression for 48 h.

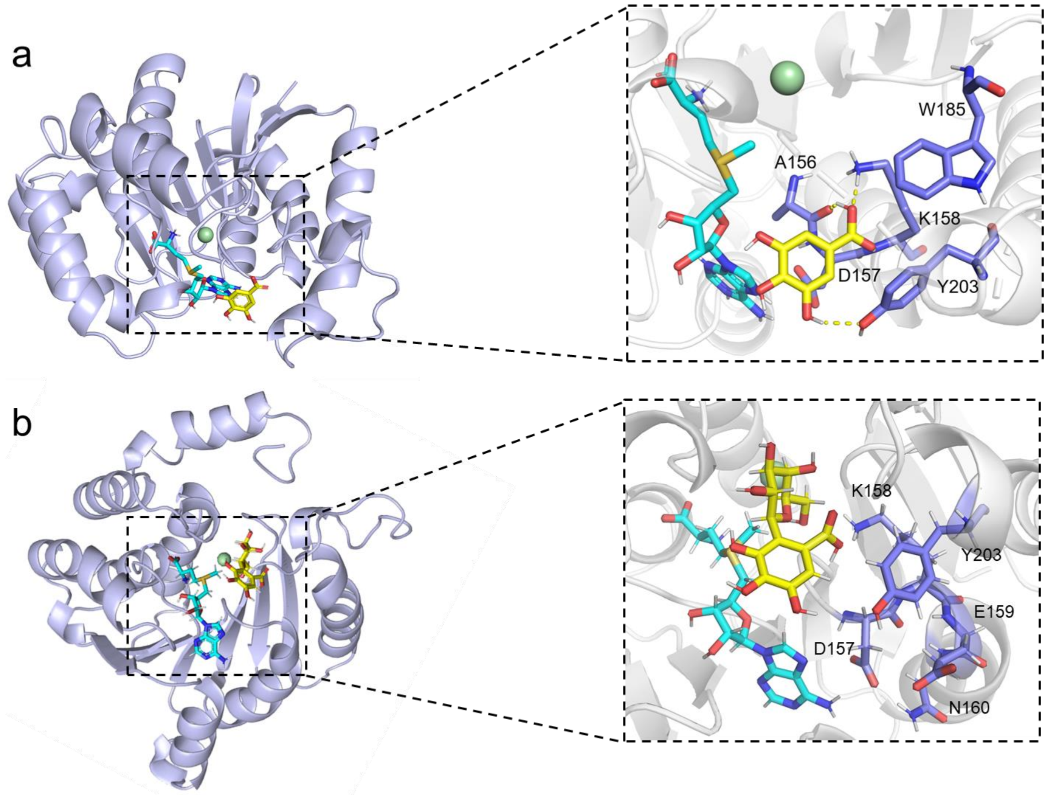

Supplementary Fig. 38. Protein modelling of AjOMT2. The protein was modelled using sorghum caffeoyl-CoA *O*-methyltransferase as a template. GA (**2**) and gallic acid 2-*C*-*β*-D-glucoside (GA-Glc, **5**) were docked into the active sites using Autodock Vina. Substrates and SAM are shown in yellow and green respectively. a) AjOMT2 substrate-binding pockets for GA (**2**), surrounding amino acids hosting GA (**2**) in the active pocket of AjOMT2. b) Substrate binding pockets of AjOMT2 for GA-Glc (**5**), amino acids surrounding GA-Glc (**5**) in the active pocket of AjOMT2.

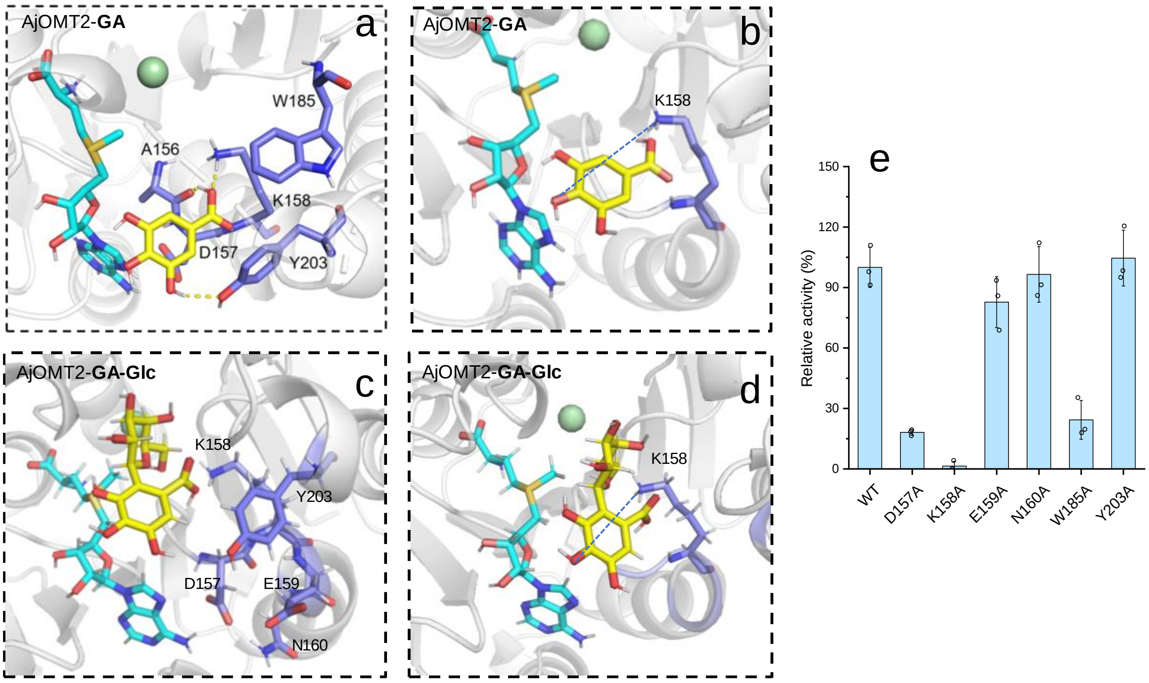

Supplementary Fig. 39. Alanine-scanning of the candidate active sites of AjOMT2. a) the candidate active sites around GA (**2**); b) the potential site acting as a catalytic base in catalyzing GA (**2**); c) the candidate active sites around the GA-Glc (**5**); d) the potential site acting as a catalytic base in catalyzing GA-Glc (**5**); e) The *in vitro* catalytic activity of the WT and the mutants of AjOMT2 was investigated with GA (**2**) as an acceptor (n=3 samples). Bars of charts represent mean ±SD of three biologically independent experiments. Source data are provided as a Source Data file.

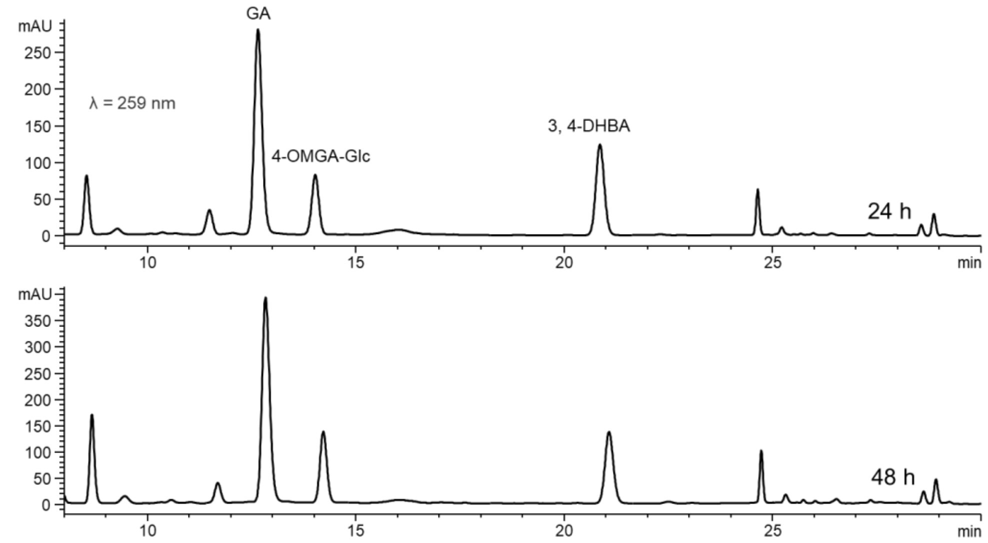

Supplementary Fig. 40. The fermentation broth supernatants of strain D4 with shake flasks were analyzed by HPLC after inducing expression for 24 h and 48 h. GA was accumulated in the fermentation with shake flasks.

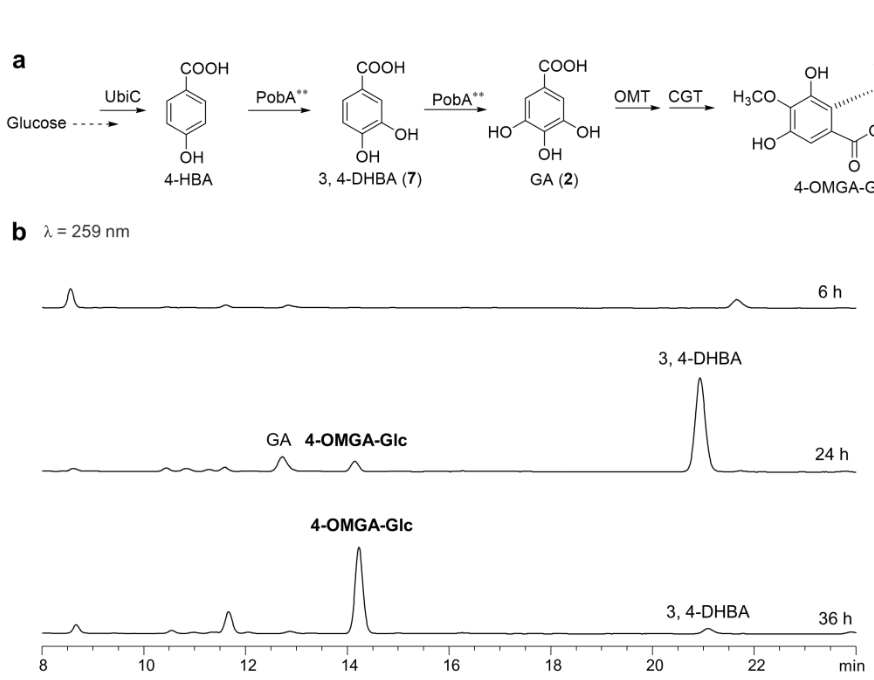

Supplementary Fig. 41. HPLC analysis of the production of 4-OMGA-Glc (4) in bioreactor at different time periods. a) The artificial biosynthetic pathway to 4-OMGA-Glc from glucose in engineered strain D4; b) HPLC analysis of the fermentation broth supernatants of strain D4 in bioreactor at different time periods (6h, 24h and 36h).

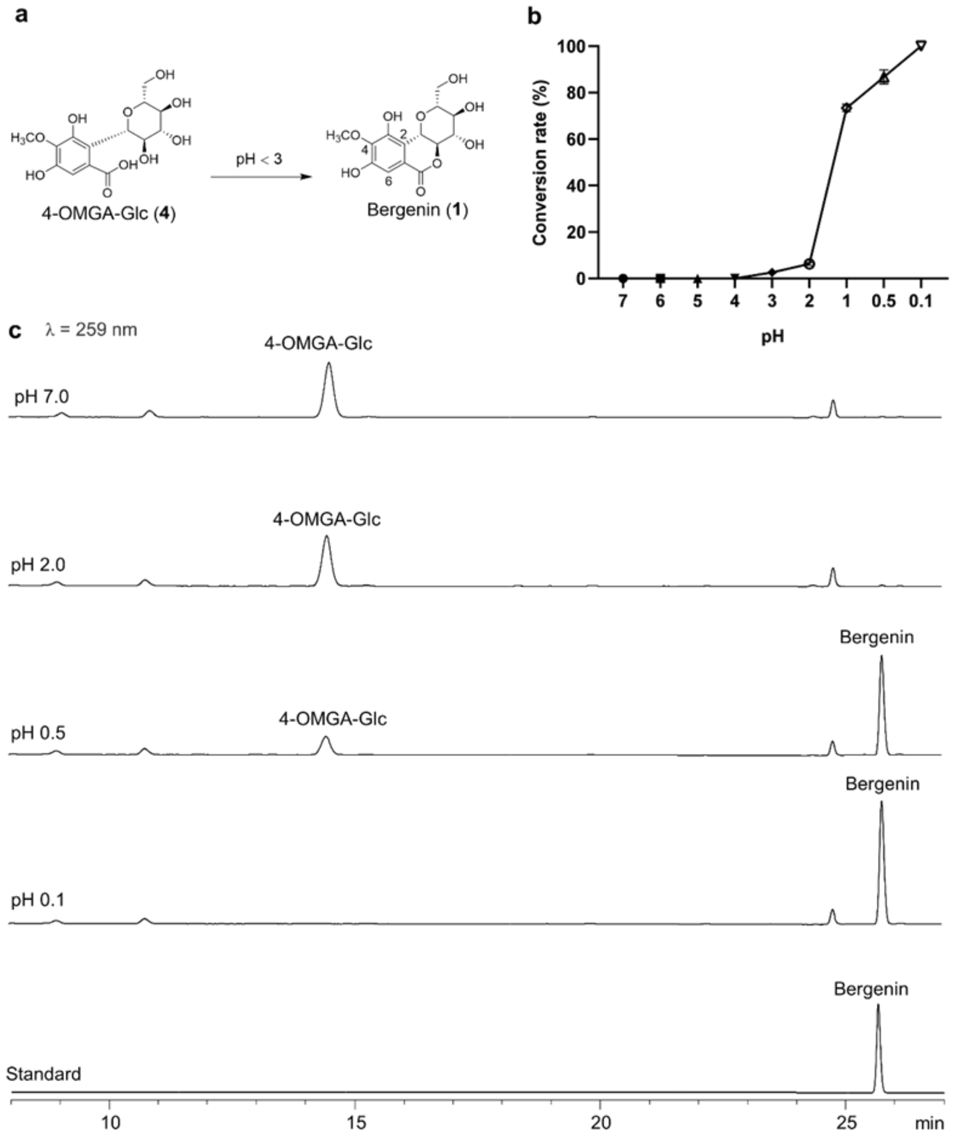

Supplementary Fig. 42. The conversion of 4-OMGA-Glc (4) into bergenin (1) by pH adjustment of the cultures. a) The reaction of 4-OMGA-Glc (**4**) in the acid environment; b) The conversion rates of 4-OMGA-Glc (**4**) at different pH values (n=3 samples); c) HPLC analysis of the conversion of 4-OMGA-Glc (**4**) in the cultures at different pH values. Line charts represent mean ±SD of three biologically independent experiments.

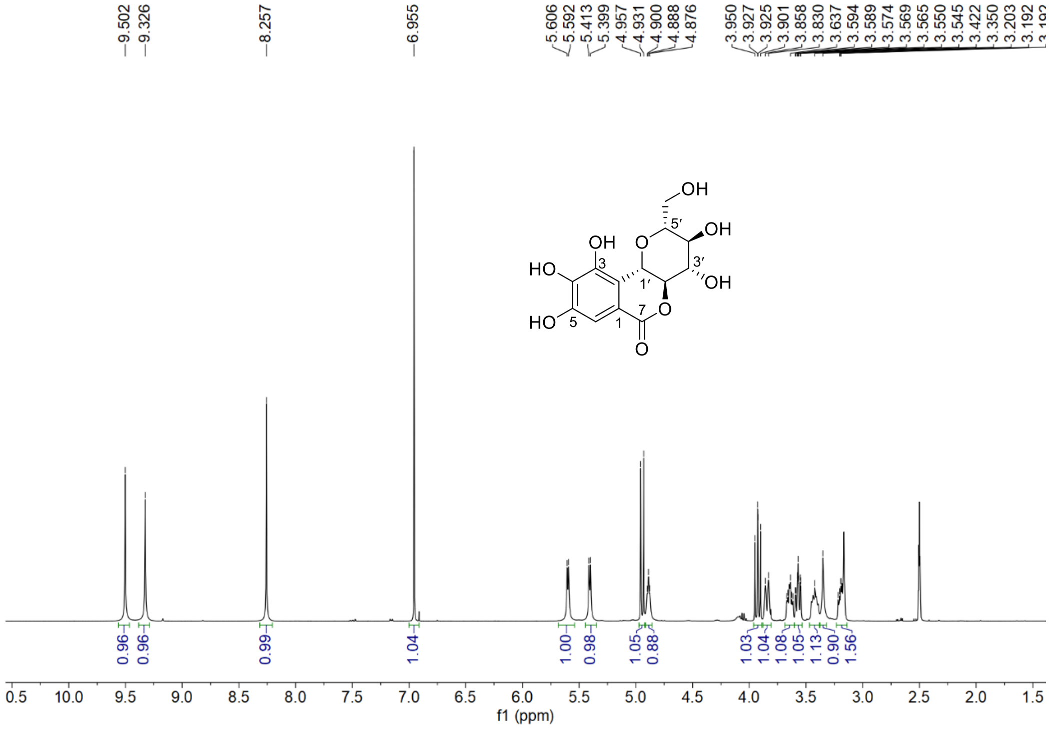

Supplementary Fig. 43. ^{1}H NMR spectrum of norbergenin (6, DMSO-d_6, 400 MHz).

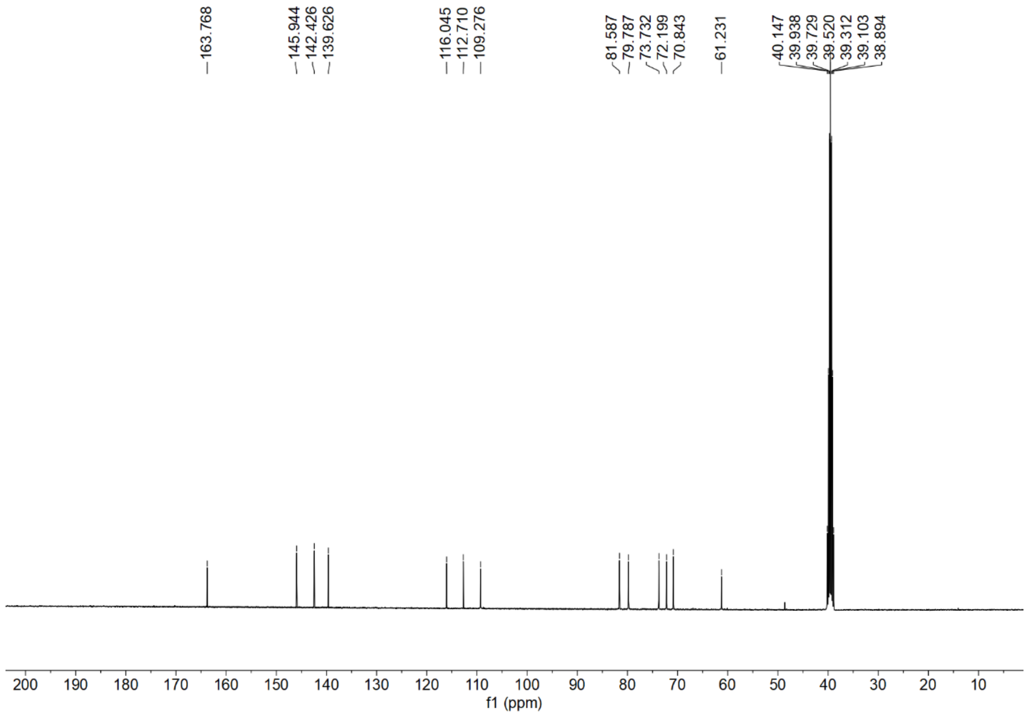

Supplementary Fig. 44. ^{13}C NMR spectrum of norbergenin (6, DMSO-d_6, 100 MHz).

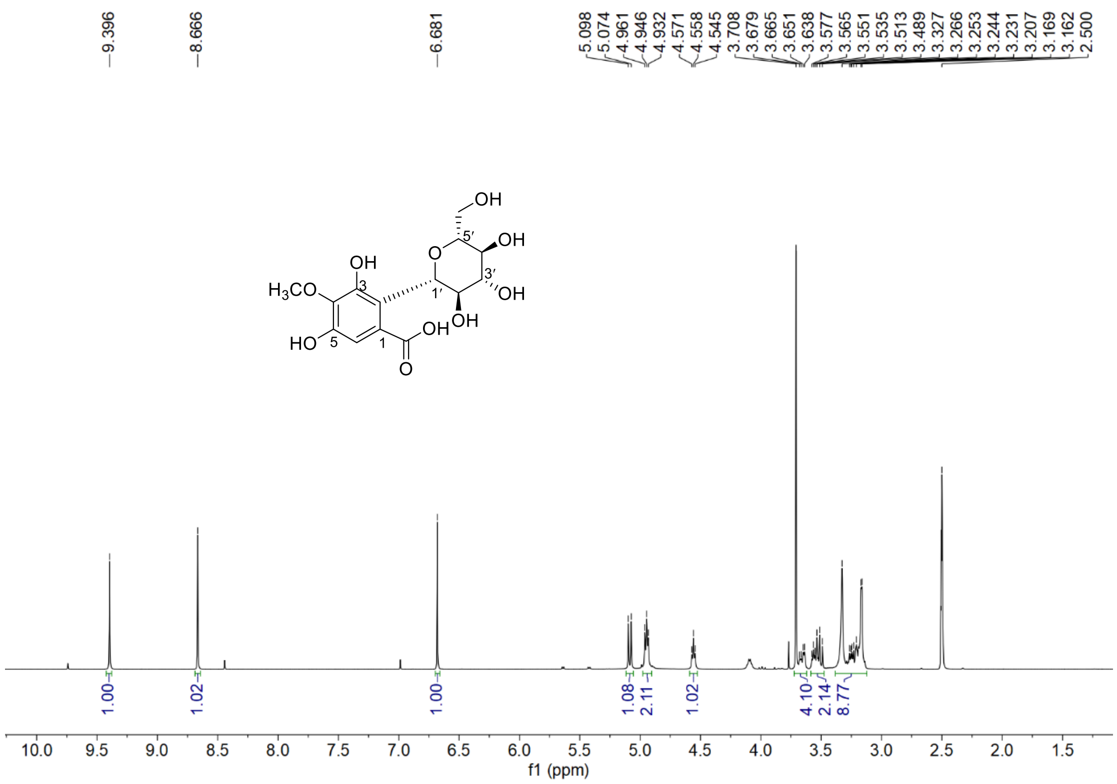

Supplementary Fig. 45. ^{1}H NMR spectrum of 4-*O*-methyl gallic acid 2-*C*-*β*-D-glycoside (4, DMSO-d_6, 400 MHz).

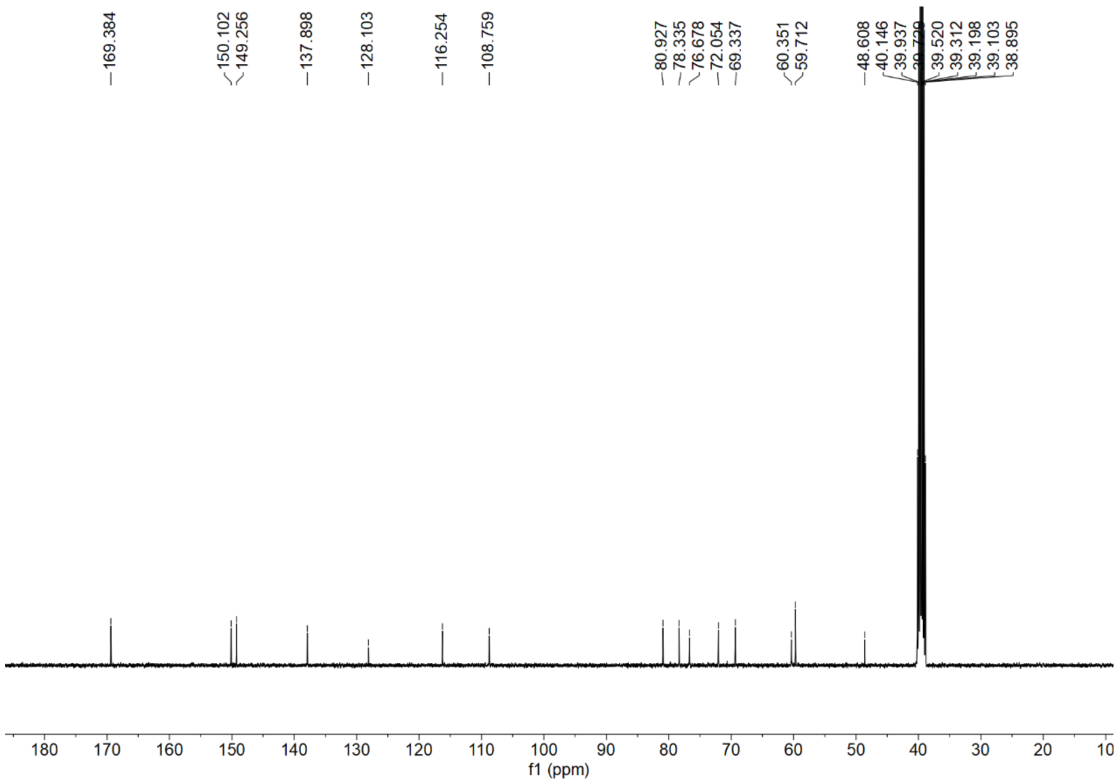

Supplementary Fig. 46. ^{13}C NMR spectrum of 4-*O*-methyl gallic acid 2-*C*-*β*-D-glycoside (4, DMSO-*d*$_6$, 100 MHz).

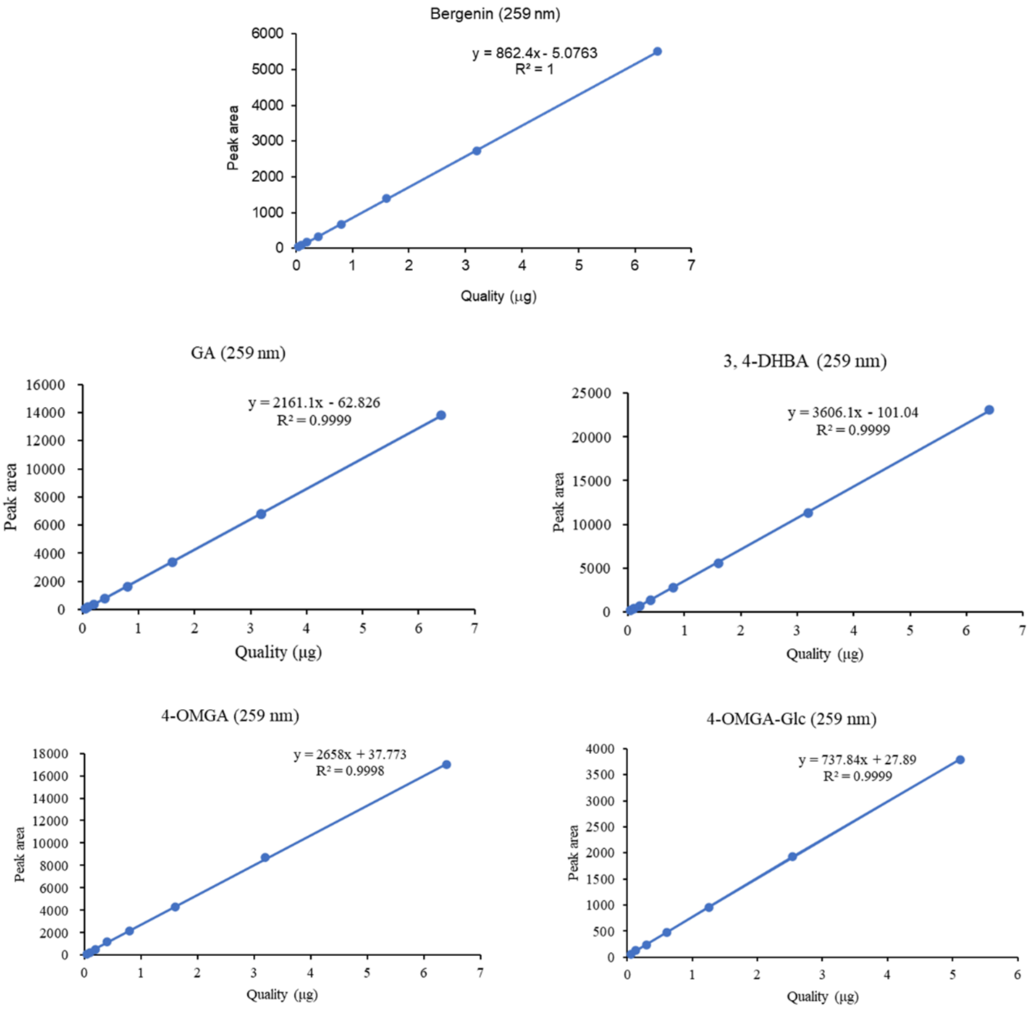

Supplementary Fig. 47. Standard curves of bergenin, GA, 3,4-DHBA, 4-OMGA, 4-OMGA-Glc. Data points represent mean of three biologically independent experiments. All data represent the means of three parallel experiments. Source data are provided as a Source Data file.

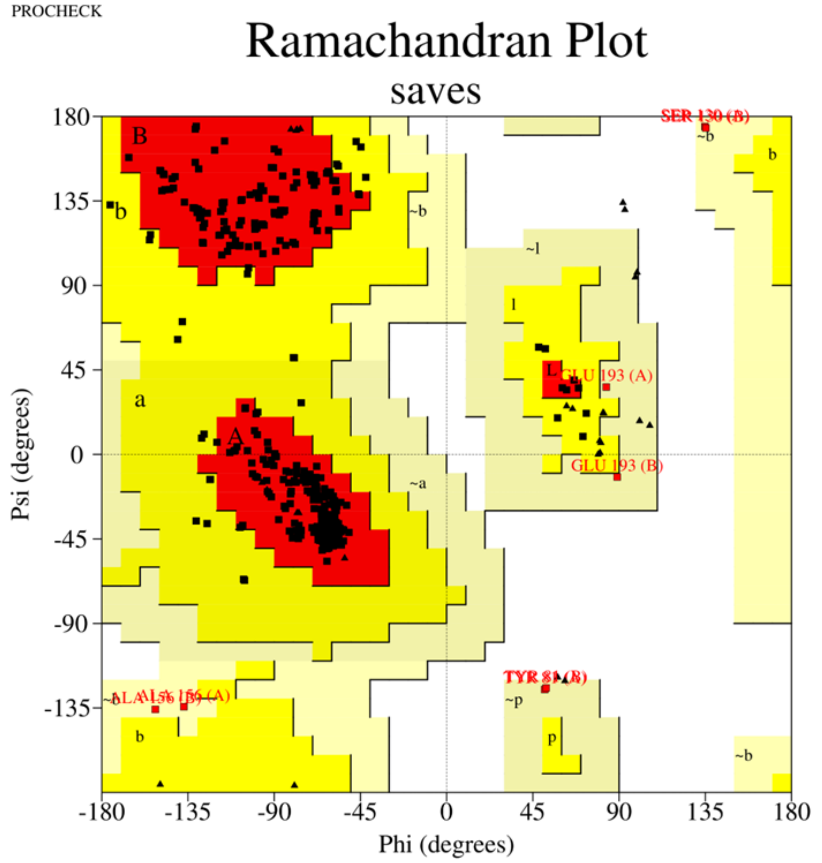

Plot statistics

Residues in most favoured regions [A,B,L]	363	91.9%
Residues in additional allowed regions [a,b,l,p]	24	6.1%
Residues in generously allowed regions [~a,~b,~l,~p]	8	2.0%
Residues in disallowed regions	0	0.0%
	----	------
Number of non-glycine and non-proline residues	395	100.0%
Number of end-residues (excl. Gly and Pro)	6	
Number of glycine residues (shown as triangles)	28	
Number of proline residues	24	

Total number of residues	453	

Based on an analysis of 118 structures of resolution of at least 2.0 Angstroms and R-factor no greater than 20%, a good quality model would be expected to have over 90% in the most favoured regions.

saves_01.ps

Supplementary Fig. 48. The Ramachandran plot of AjOMT2.

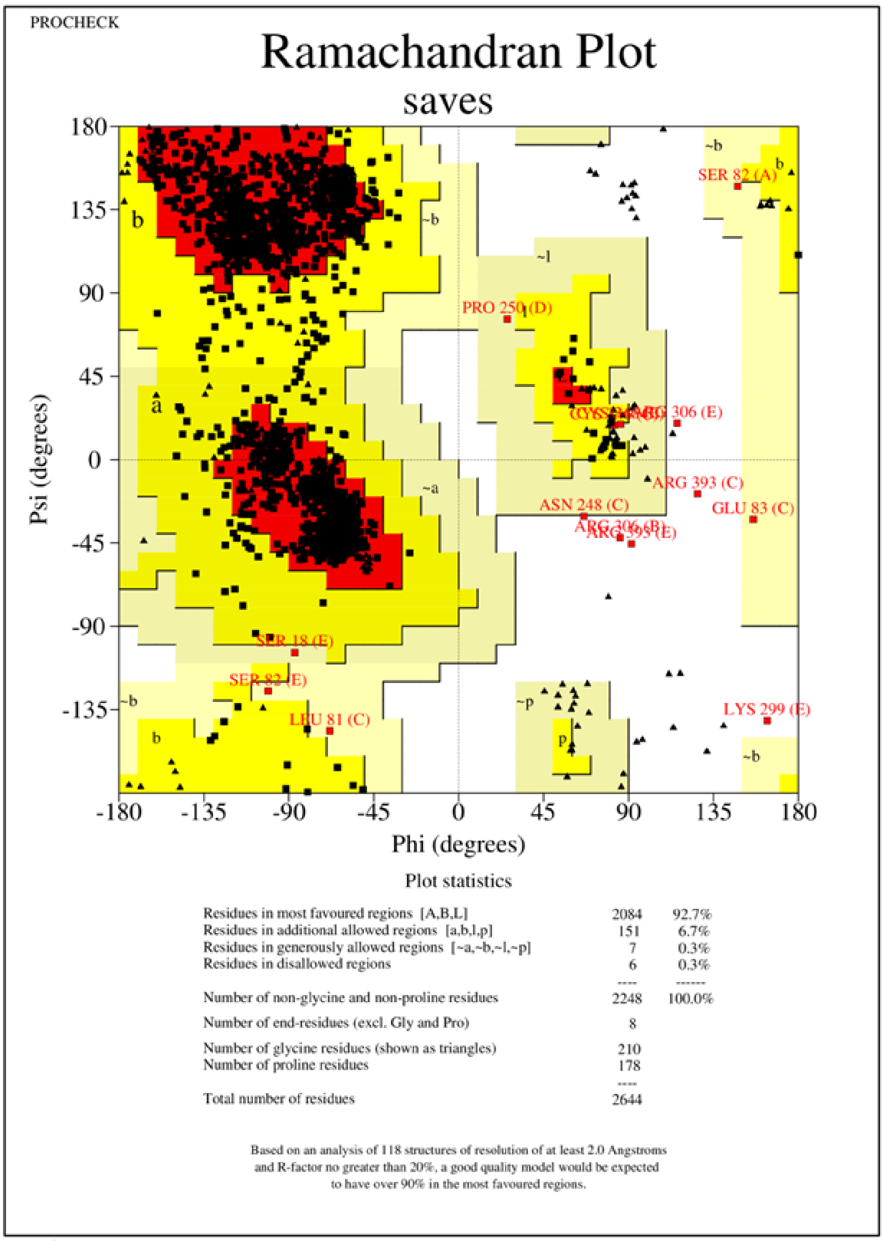

saves_01.ps

Supplementary Fig. 49. The Ramachandran plot of AjCGT1.

Supplementary references

1. Taneyama, M., Yoshida, S., Kobayashi, M. & Hasegawa, M. Isolation of norbergenin from *Saxifraga stolonifera*. *Phytochemistry*, **22**, 1053−1054 (1983).

2. Brazier-Hicks, M., *et al*. The *C*-glycosylation of flavonoids in cereals. *J. Biol. Chem.*, **284**, 17926–17934 (2009).

3. Ito, T., Fujimoto, S., Suito, F., Shimosaka, M. & Taguchi, G. *C*-glycosyltransferases catalyzing the formation of di-*C*-glucosyl flavonoids in citrus plants. *Plant J.*, **91,** 187–198 (2017).

4. Liu, X., *et al*. Characterization of a caffeoyl-CoA *O*-methyltransferase like enzyme involved in biosynthesis of polymethoxylated flavones in *Citrus reticulata*. *J. Exp. Bot.*, **71**, 3066–3079 (2020).

5. Widiez, T., *et al*. Functional characterization of two new members of the caffeoyl CoA *O*-methyltransferase-like gene family from *Vanilla planifolia* reveals a new class of plastid-localized *O*-methyltransferases. *Plant Mol. Biol.,* **76**, 475–488 (2011).
