# Supplementary material for: Unravelling and reconstructing the biosynthetic pathway of bergenin
Source: Nat Commun. 2024 Apr 26;15:3539. doi: 10.1038/s41467-024-47502-2 (PMC11053098; doi:10.1038/s41467-024-47502-2)
Supplement: Supplementary file 3 — Description of Additional Supplementary Files [file 41467_2024_47502_MOESM3_ESM.pdf]

Description of Additional Supplementary Files

File Name: Supplementary Data 1
Description: Primer pairs used in vectors construction

File Name: Supplementary Data 2
Description: Plasmids used in this study
